# A Multitarget Approach against Neuroinflammation: Alkyl Substituted Coumarins as Inhibitors of Enzymes Involved in Neurodegeneration

**DOI:** 10.3390/antiox12122044

**Published:** 2023-11-25

**Authors:** Emanuela Berrino, Simone Carradori, Fabrizio Carta, Francesco Melfi, Marialucia Gallorini, Giulio Poli, Tiziano Tuccinardi, José G. Fernández-Bolaños, Óscar López, Jacobus P. Petzer, Anél Petzer, Paolo Guglielmi, Daniela Secci, Claudiu T. Supuran

**Affiliations:** 1 Department of Drug Chemistry and Technologies, Sapienza University of Rome, P.le A. Moro 5, 00185 Rome, Italy; emanuela.berrino@uniroma1.it (E.B.); paolo.guglielmi@uniroma1.it (P.G.); daniela.secci@uniroma1.it (D.S.); 2 NEUROFARBA Department, Sezione di Scienze Farmaceutiche e Nutraceutiche, Università degli Studi di Firenze, Via Ugo Schiff 6, 50019 Florence, Italy; fabrizio.carta@unifi.it (F.C.); claudiu.supuran@unifi.it (C.T.S.); 3 Department of Pharmacy, ‘‘G. d’Annunzio” University of Chieti-Pescara, via dei Vestini 31, 66100 Chieti, Italy; francesco.melfi@unich.it (F.M.); marialucia.gallorini@unich.it (M.G.); 4 Department of Pharmacy, University of Pisa, Via Bonanno 6, 56126 Pisa, Italy; giulio.poli@unipi.it (G.P.); tiziano.tuccinardi@unipi.it (T.T.); 5 Departamento de Química Orgánica, Facultad de Química, Universidad de Sevilla, Apartado 1203, 41012 Seville, Spain; bolanos@us.es (J.G.F.-B.); osc-lopez@us.es (Ó.L.); 6 Pharmaceutical Chemistry, School of Pharmacy and Centre of Excellence for Pharmaceutical Sciences, North-West University, Potchefstroom 2531, South Africa; jacques.petzer@nwu.ac.za (J.P.P.); 12264954@nwu.ac.za (A.P.)

**Keywords:** carbonic anhydrase, inhibitors, monoamine oxidase (MAO), coumarin, cholinesterase, Parkinson’s disease, neuroinflammation

## Abstract

Neurodegenerative disorders (NDs) include a large range of diseases characterized by neural dysfunction with a multifactorial etiology. The most common NDs are Alzheimer’s disease and Parkinson’s disease, in which cholinergic and dopaminergic systems are impaired, respectively. Despite different brain regions being affected, oxidative stress and inflammation were found to be common triggers in the pathogenesis and progression of both diseases. By taking advantage of a multi-target approach, in this work we explored alkyl substituted coumarins as neuroprotective agents, capable to reduce oxidative stress and inflammation by inhibiting enzymes involved in neurodegeneration, among which are Carbonic Anhydrases (CAs), Monoamine Oxidases (MAOs), and Cholinesterases (ChEs). The compounds were synthesized and profiled against the three targeted enzymes. The binding mode of the most promising compounds (**7** and **9**) within MAO-A and -B was analyzed through molecular modeling studies, providing and explanation for the different selectivities observed for the MAO isoforms. In vitro biological studies using LPS-stimulated rat astrocytes showed that some compounds were able to counteract the oxidative stress-induced neuroinflammation and hamper interleukin-6 secretion, confirming the success of this multitarget approach.

## 1. Introduction

Neurodegenerative disorders (NDs) refer to pathological states associated with neurodegeneration. Depending on the affected brain area, different clinical findings can be observed in such patients. Alzheimer’s and Parkinson’s diseases are the most common neurodegenerative disorders, characterized by impairment of cholinergic and dopaminergic brain activities, respectively [1,2]. These extremely disabling pathologies, for which there are still no effective cures, are characterized by a multifactorial etiology [1,3]. In this context, the therapy of neurodegenerative disorders could take advantage of multi-target directed ligands (MTDLs) which are able to affect impaired cellular mechanisms of these conditions [4,5,6,7]. In the light of the role that human monoamine oxidase B (MAO-B) as well as acetylcholinesterase and butyrylcholinesterase (AChE and BuChE) play in neurodegenerative disorders, MTDLs are often directed to these targets [8,9,10]. For example, compounds endowed with MAO-B as well as AChE and BuChE inhibitory activities have been proposed as useful candidates for Alzheimer’s disease treatment. Similarly, molecules with MAO-B inhibitory activity combined with antioxidant properties might be exploited to develop novel compounds for the treatment of Parkinson’s disease [11,12]. Human monoamine oxidases (hMAOs) are mitochondrial bound flavoenzymes which catalyze the oxidative catabolism of amines [13,14]. Two different isoforms called hMAO-A and hMAO-B have been described in human. These isoforms are similar, sharing ~70% sequence identity; however, some differences in the active sites have been recognized and often exploited to obtain isoform selective inhibitors [13,15]. The physio-pathological roles of hMAO-A and hMAO-B have been widely explored along with their different tissue distribution. hMAOs are co-expressed in almost all human organs except for placenta, where hMAO-A is predominant, and platelets and lymphocytes are mostly expressing the B-isoform [13,15]. In addition, hMAO-A is abundant in the intestinal tract, whereas hMAO-B is detected in the brain and liver. This characteristic makes hMAO-B an exploitable target in ND treatment, where the inhibition of this isoform increases neurotransmission (mainly dopaminergic transmission) and reduces the production of hydrogen peroxide (H_2_O_2_), a by-product of the regeneration of the functional form of the MAO cofactor (FAD) [16,17]. Indeed, enhanced expression or abnormal activity of hMAOs leads to high generation of H_2_O_2_ which could exert oxidative damage. The presence of cations (e.g., iron and copper) stimulate the production of H_2_O_2_-derived reactive oxygen species (ROS) through the well-known Fenton reaction. ROS are less stable than H_2_O_2_ and react readily with biomolecules causing structural/functional damage and cell death. Cellular stress caused by MAO has been associated with different pathologies spanning from neurodegenerative disorders to cardiomyopathies [16,18,19].

Cholinesterases are responsible for the enzymatic cleavage of the neurotransmitter acetylcholine (ACh) [20,21]. Acetylcholinesterase, the focus of the symptomatic therapy of AD, is located in both the central and peripheral nervous systems and in the muscular motor plaques. Besides the symptomatic actions, AChE inhibitors were found also to be neuroprotective [22,23,24]. Butyrylcholinesterase has been found to be upregulated in brain and peripheral tissues upon advanced AD [25]. Two different regions of the AChE enzyme have been recognized as preferred binding sites for inhibitors: the catalytic active site (CAS) is at the bottom of the active site cleft which contains the catalytic triad (Ser-His-Glu) responsible for ACh cleavage, whereas the peripheral anionic binding site (PAS) is at the entrance of the active site gorge, which is involved in the allosteric modulation of catalysis.

In the last years, the role of some carbonic anhydrase (CA) isoforms in the field of ND has been recognized, as some of them were found to be involved in the regulation of oxidative stress and progression of neurodegenerative disorders [26]. CAs are ubiquitous enzymes and catalyze the reversible hydration of carbon dioxide. Until now, eight unrelated genetic CA families have been described [27]. Human (h) carbonic anhydrases belong to the α-family and fifteen isoforms have been discovered and analyzed for their enzymatic activity, tissue and organ distribution, as well as physiological/pathological roles. Therefore, the possible therapeutic applications of CA inhibitors are numerous [28]. CA inhibitors are currently licensed for the cure of glaucoma, hypertension, altitude sickness, epilepsy, and obesity and are considered validated targets for the treatment of hypoxic tumors, with a compound (SLC-0111) completing Phase Ib/II trials for advanced metastatic solid tumors [28,29]. Although different in terms of substrate specificity, functions, and tissue/organ distribution, these enzymes share common inhibitors, e.g., coumarins, naturally occurring compounds which were found to inhibit CA enzyme through an unconventional mechanism especially if not hindered around the lactone portion [30,31]. Inhibition was found to occur through the occlusion of the enzyme active site entrance when the coumarin derivative was in the hydrolyzed form, the hydroxy-cinnamic acid, generated by the esterase activity of CA itself [31]. Interestingly, the outer region of the binding site cavity, where coumarins were found to bind the enzyme, is the most variable among the 15 human expressed isoforms. Moreover, some CA isoenzymes show diverse esterase activity because of structural differences between their active sites. These features can be exploited to obtain selectivity of coumarins towards specific isoforms so that, unlike sulfonamide compounds which are pan-CA inhibitors, coumarins are classified as more selective inhibitors, with particular affinity for the isoforms IX and XII [31].

**Figure 1:**
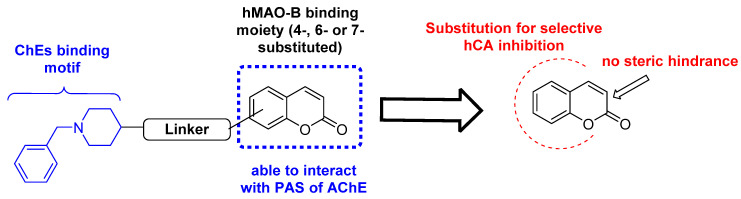
Rational design of substituted coumarins as pleiotropic inhibitors of enzymes involved in neurodegenerative disorders.

Coumarins are privileged scaffolds in medicinal chemistry thanks to their favorable physicochemical properties as well as the presence of several positions that are suitable for substitution on the coumarin the nucleus. This allows for the synthesis of a large variety of synthetic derivatives with numerous biological activities reported to date [32,33]. Among the well-recognized activities, antitumor, anti-inflammatory, antioxidant, antimicrobial, antiviral, and antidepressant activities have been reported [32,34,35,36]. Coumarin-based compounds may be particularly appropriate for the treatment of neurodegenerative disorders (NDs), considering the multifactorial etiology of such diseases and the possibility to modulate with a singular molecular entity different biological target involved in their progression [6,33]. In this regard, differently substituted coumarin derivatives have been designed to act as hMAO and ChE inhibitors [37,38,39,40,41,42,43,44,45,46]. The design of these inhibitors was usually based on the combination of molecular attributes required for hMAO-B (4-, 6- or 7-substitution) and AChE/BuChE inhibition, respectively. For example, the coumarin core endowed with hMAO-B inhibitory activity was substituted with the benzylpiperidine moiety that is present in donepezil, a well-known AChE inhibitor. However, the coumarin scaffold may interact with PAS of AChE, in turn involved in the self-aggregation of β-amyloid plaques, thus justifying its use for MTDL development (Figure 1).

Despite the potential of coumarins in the treatment of NDs and the increasing evidence of CA involvement in such pathological states, most of the compounds studied thus far in ND models are sulfonamide-based compounds (acetazolamide, AAZ, and methazolamide, MTZ) [26]. There is a large amount of literature describing the potential of coumarins for the treatment of tumor- and inflammation-based diseases [47,48,49,50], which is based on the potent and selective action usually reported for coumarins against the CA IX and XII, involved in tumor progression [51]. However, these isoforms are also expressed in the CNS, although with different distributions, and they are known to be involved in the modulation of inflammatory conditions [26]. As suggested by recent evidence, CA inhibition could be protective to neuro-vascular cells during the development of ND diseases, which are characterized by several cellular dysfunctions, oxidative stress, and the release of inflammatory signaling molecules [26,52]. Nevertheless, studies exploring substituted coumarin compounds for the treatment of NDs only evaluated their MAO and ChE inhibitory properties, without profiling them against CA isoforms [37,39,40]. In this context, astrocytes are the most abundant cells in the CNS, being also the most heterogeneous under homeostatic conditions. It has been indeed reported that they display different molecular and morphological responses. In this respect, ‘astrocytosis’ or ‘reactive gliosis’ contributes to tissue repair and promote CNS pathology in the context of trauma, infection, and neurodegenerative diseases [53,54].

Thus, this approach is the first to comprehensively investigate the potential neuroprotective activities of the “pleiotropic” coumarin scaffold involving not only carbonic anhydrase inhibition, after the demonstration of the dual inhibitory activity (MAOs and Cas) exerted by other substituted coumarins [55] (Figure 1).

## 2. Methods and Materials

**General synthetic procedure** [56]**:** The starting material (0.5 g, 1.0 eq.) in dry DMF (4 mL), K_2_CO_3_ (1.5 eq.) was added, and the mixture stirred for 15 min at r.t. The appropriate alkyl halide (2.0 eq.) was subsequently reacted dropwise. The reaction was stirred at r.t. or 150 °C, based on the alkyl halide type, till consumption of starting material by TLC visualization. The reaction mixture was quenched with ice (slushed), which provided a thick precipitate that was collected by filtration and dried.

**4-(Pent-4-yn-1-yloxy)-2*H*-chromen-2-one (1).** Synthesized using 4-hydroxy-2*H*-chromen-2-one as starting material and 5-chloropent-1-yne as alkyl halide. Reaction performed at 150 °C. Compound **1** collected as a white powder: 80% yield; m.p. 120–122 °C; silica gel TLC R*f* 0.65 (EtOAc/n-Hex 30% *v*/*v*); δ_H_ (400 MHz, DMSO-*d*_6_) 2.01 (2H, *p*, *J* = 6.6, C*H*_2_), 2.42 (2H, td, *J* = 7.1, 2.7, C*H*_2_), 2.85 (1H, t, *J* = 2.7, C*H*), 4.28 (2H, t, *J* = 6.0, C*H*_2_), 5.91 (1H, s, Ar-*H*), 7.41 (2H, m, 2 x Ar-*H*), 7.66 (1H, t, *J* = 7.8, Ar-*H*), 7.86 (1H, d, *J* = 7.9, Ar-*H*); δ_C_ (100 MHz, DMSO-*d*_6_) 14.5, 27.0, 68.0, 71.8, 83.4, 90.5, 115.1, 116.4, 123.0, 124.1, 132.7, 152.7, 161.6, 164.8.

**4-(Hex-5-yn-1-yloxy)-2*H*-chromen-2-one (2).** Synthesized using 4-hydroxy-2*H*-chromen-2-one as starting material and 6-chlorohex-1-yne as alkyl halide. Reaction performed at 150 °C. Compound **2** collected as a white powder: 80% yield; m.p. 120–122 °C; silica gel TLC R*f* 0.55 (EtOAc/n-Hex 30% *v*/*v*); δ_H_ (400 MHz, DMSO-*d*_6_) 1.70 (2H, m, C*H*_2_), 1.95 (3H, m, C*H*_2_ and C*H*), 2.31 (2H, m, C*H*_2_), 4.15–4.18 (2H, t, *J* = 6.2, C*H*_2_), 5.66 (1H, s, Ar-*H*), 7.39 (1H, d, *J* = 7.8, Ar-*H*), 7.43 (1H, d, *J* = 8.1, Ar-*H*), 7.69 (1H, t, *J* = 8.1, Ar-*H*), 7.85 (1H, d, *J* = 7.8, Ar-*H*); δ_C_ (100 MHz, DMSO-*d*_6_) 17.3, 24.5, 27.0, 68.9, 71.4, 84.1, 90.5, 115.2, 116.4, 122.7, 124.1, 132.6, 152.7, 161.6, 164.9.

**6-(Prop-2-ynyloxy)-2*H*-chromen-2-one (3).** Synthesized using 6-hydroxy-2*H*-chromen-2-one as starting material and propargyl bromide 80% in toluene as alkyl halide at r.t. Compound **3** collected as a white powder: 65% yield; m.p. 165–166 °C; silica gel TLC R*f* 0.63 (EtOAc/n-Hex 50% *v*/*v*); δ_H_ (400 MHz, DMSO-*d*_6_) 3.60 (1H, t, *J* = 2.4, C*H*), 4.86 (2H, d, *J* = 2.4, C*H*_2_), 6.50 (1H, d, *J* = 9.6, Ar-*H*), 7.26 (1H, dd, *J* = 9.0, 2.9, Ar-*H*), 7.34 (1H, d, *J* = 2.9, Ar-*H*), 7.38 (1H, d, *J* = 9.0, Ar-*H*), 8.02 (1H, d, *J* = 9.6, Ar-*H*); δ_C_ (100 MHz, DMSO-*d*_6_) 56.0, 78.6, 78.9, 112.3, 116.8, 117.4, 119.2, 120.0, 144.0, 148.3, 153.4, 160.1. Experimental in agreement with reported data [57].

**6-(But-2-yn-1-yloxy)-2*H*-chromen-2-one (4).** Synthesized using 6-hydroxy-2*H*-chromen-2-one as starting material and 1-bromobut-2-yne as alkyl halide at r.t. Compound **4** collected as a white powder: 84% yield; m.p; 157–159 °C; silica gel TLC R*f* 0.75 (EtOAc/n-Hex 30% *v*/*v*); δ_H_ (400 MHz, DMSO-*d*_6_) 1.83 (3H, t, *J* = 2.3, C*H*_3_), 4.79 (2H, q, *J* = 2.4, C*H*_2_), 6.49 (1H, d, *J* = 9.6, Ar-*H*), 7.23 (1H, dd, *J* = 9.0, 3.0 Ar*-H*), 7.31 (1H, d, *J* = 3.0, Ar-*H*), 7.36 (1H, t, *J* = 9.0, Ar-*H*), 8.01 (1H, d, *J* = 9.6, Ar-*H*); δ_C_ (100 MHz, DMSO-*d*_6_) 3.0, 56.5, 74.4, 83.9, 112.2, 116.6, 117.3, 119.1, 119.9, 143.9, 148.1, 153.6, 160.0.

**7-(Prop-2-ynyloxy)-2*H*-chromen-2-one (5).** Synthesized using 7-hydroxy-2*H*-chromen-2-one as starting material and propargyl bromide 80% in toluene as alkyl halide at r.t.. Compound **5** collected as a white powder: 73% yield; δ_H_ (400 MHz, DMSO-*d*_6_) 3.65 (1H, t, *J* = 2.4, C*H*), 4.94 (2H, d, *J* = 2.4, C*H*_2_), 6.32 (1H, d, *J* = 9.4, Ar-*H*), 7.00 (1H, dd, *J* = 8.6, 2.5, Ar-*H*), 7.05 (1H, d, *J* = 2.5, Ar-*H*), 7.66 (1H, d, *J* = 8.6, Ar-*H*), 8.00 (1H, d, *J* = 9.5, Ar-*H*). Experimental analyses in agreement with reported data [57].

**7-(But-2-yn-1-yloxy)-2*H*-chromen-2-one (6).** Synthesized using 7-hydroxy-2*H*-chromen-2-one as starting material and 1-bromobut-2-yne as alkyl halide at r.t. Compound **6** collected as a white powder: 63% yield; m.p. 130–132 °C; silica gel TLC R*f* 0.70 (EtOAc/n-Hex 30% *v*/*v*); δ_H_ (400 MHz, DMSO-*d*_6_) 1.88 (3H, s, C*H*_3_), 4.91 (2H, m, C*H*_2_), 6.31 (1H, d, *J* = 9.5, Ar-*H*), 7.01 (1H, d, *J* = 8.6, Ar-*H*), 7.05 (1H, s, Ar-*H*), 7.64 (1H, d, *J* = 8.6, Ar-*H*), 7.99 (1H, d, *J* = 9.5, Ar-*H*); δ_C_ (100 MHz, DMSO-*d*_6_) 3.0, 56.6, 73.9, 84.3, 101.6, 112.7, 112.8, 112.9, 129.4, 144.2, 155.1, 160.1, 160.4.

**7-(Pent-2-yn-1-yloxy)-2*H*-chromen-2-one (7).** Synthesized using 7-hydroxy-2*H*-chromen-2-one as starting material and 1-bromopent-2-yne as alkyl halide at r.t. Compound **7** collected as a white powder: 77% yield; m.p. 140–142 °C; silica gel TLC R*f* 0.75 (EtOAc/n-Hex 40% *v*/*v*); δ_H_ (400 MHz, DMSO-*d*_6_) 1.06 (3H, t, *J* = 7.5, C*H*_3_), 2.24 (2H, qt, *J* = 7.5, 2.1, C*H*_2_), 4.88 (2H, s, C*H*_2_), 6.31 (1H, d, *J* = 9.5, Ar-*H*), 6.97 (1H, dd, *J* = 8.6, 2.5, Ar-*H*) 7.02 (1H, d, *J* = 2.4, Ar-*H*) 7.64 (1H, d, *J* = 8.6, Ar-*H*) 7.99 (1H, d, *J* = 9.5, Ar-*H*); δ_C_ (100 MHz, DMSO-*d*_6_) 11.6, 13.4, 56.6, 74.1, 89.7, 101.6, 112.7, 112.8, 112.9, 129.4, 144.2, 155.1, 160.1, 160.4.

**7-(Pent-4-yn-1-yloxy)-2*H*-chromen-2-one (8).** Synthesized using 7-hydroxy-2*H*-chromen-2-one as starting material and 5-chloropent-1-yne as alkyl halide at 150 °C. Compound **8** collected as a white powder: 71% yield; m.p. 160–162 °C; silica gel TLC R*f* 0.35 (EtOAc/n-Hex 40% *v*/*v*); δ_H_ (400 MHz, DMSO-*d*_6_) 1.92 (2H, *p*, *J* = 6.6, C*H*_2_), 2.34 (2H, td, *J* = 7.1, 2.7, C*H*_2_), 2.81 (1H, t, *J* = 2.7, C*H*), 4.14 (2H, t, *J* = 6.2, C*H*_2_), 6.28 (1H, d, *J* = 9.5, Ar-*H*), 6.95 (1H, dd, *J* = 8.6, 2.4, Ar-*H*), 6.99 (1H, d, *J* = 2.4, Ar-*H)*, 7.62 (1H, d, *J* = 8.5, Ar-*H*), 7.98 (1H, d, *J* = 9.5, Ar-*H*); δ_C_ (100 MHz, DMSO-*d*_6_) 14.4, 27.4, 66.7, 71.7, 83.5, 101.2, 112.4, 112.5, 112.6, 129.5, 144.2, 155.4, 160.2, 161.6.

**7-(Hex-5-yn-1-yloxy)-2*H*-chromen-2-one (9).** Synthesized using 7-hydroxy-2*H*-chromen-2-one as starting material and 6-chlorohex-1-yne as alkyl halide at 150 °C. Compound **9** collected as a white powder: 80% yield; m.p. 150–152 °C; silica gel TLC R*f* 0.66 (EtOAc/n-Hex 40% *v*/*v*); δ_H_ (400 MHz, DMSO-*d*_6_) 1.61 (2H, *p*, *J* = 7.3, C*H_2_*), 1.83 (2H, *p*, *J* = 6.7, C*H_2_*), 2.24 (2H, td, *J* = 7.1, 2.7, C*H_2_*), 2.77 (1H, t, *J =* 2.7, C*H*), 4.10 (2H, t, *J =* 6.4, C*H_2_*), 6.28 (1H, d, *J =* 9.4, Ar-*H*), 6.94 (1H, dd, *J* = 8.5, 2.5, Ar*-H*), 6.98 (1H, d, *J* = 2.3, Ar*-H*), 7.62 (1H, d, *J* = 8.5, Ar*-H*), 7.98 (1H, d, *J* = 9.5, Ar*-H*); δ_C_ (100 MHz, DMSO-*d*_6_) 17.4, 24.5, 27.5, 67.7, 71.3, 84.2, 101.1, 112.2, 112.3, 112.6, 129.4, 144.3, 155.4, 160.2, 161.8.

### 2.1. CA Inhibition

An Applied Photophysics stopped-flow instrument measured the CA catalyzed CO_2_ hydration activity [58]. Phenol red (0.2 mM) was the indicator, with measurements taken at the absorbance maximum of 557 nm. Hepes (10 mM, pH 7.5) supplemented with 0.1 M Na_2_SO_4_ served as reaction buffer and the CA-catalyzed CO_2_ hydration reaction was monitored for 10–100 s. The CO_2_ concentrations varied from 1.7 to 17 mM. The uncatalyzed rates were also measured and subtracted. Stock solutions of inhibitors (10 mM) were provided in distilled-deionized water containing 10% of DMSO and dilutions up to 0.001 μM were prepared with the reaction buffer.

### 2.2. hMAO-A and B Inhibition

The catalytic activities of hMAO-A and hMAO-B were measured using the recombinant enzymes (Sigma-Aldrich, Modderfontain, South Africa) according to the reported procedure [59,60]. The non-selective substrate, kynuramine, was the substrate for both hMAO isoforms and is metabolized by hMAOs to 4-hydroxyquinoline, which was measured by fluorescence spectrophotometry. The hMAO enzymes and kynuramine (50 µM) were incubated in the presence of the test compounds at inhibitor concentrations ranging from 0.003 to 100 µM for 20 min. Sodium hydroxide was added to terminate the reactions and 4-hydroxyquinoline was measured at endpoint by fluorescence spectrophotometry. Sigmoidal plots of enzyme activity versus inhibitor concentration (log [I]) were plotted with Prism 5 (GraphPad, v. 5.03) from which the IC_50_ values were calculated.

Lineweaver–Burk plots were generated by analyzing the hMAO activity in the presence of compound **9** at 0 × IC_50_, ¼ × IC_50_, ½ × IC_50_, ¾ × IC_50_ and 1 × IC_50_. For each plot, the kynuramine concentrations varied from 22 to 250 μM. Linear plots of the slopes of the Lineweaver–Burk plots versus inhibitor concentration were graphed, from which the enzyme-inhibitor dissociation constants, *K*_i_, were calculated (*K*_i_ = −x when y = 0). Linear regression was carried out with Prism 5 [61].

### 2.3. AChE and BuChE Inhibition

Inhibition of AChE (electric eel) and BuChE (equine serum) was evaluated using minor modifications of the classical Ellman assay using a Thermo Scientific^TM^ Varioskan^TM^ LUX microplate reader (Thermo Fisher Scientific, Waltham, MA USA) and Greiner F-bottom 96-well plates [62]. Inhibitors were initially tested at a 100 µM concentration, using a substrate concentration equal to the *K*_M_ value of each enzyme; for those compounds that exhibited inhibition higher than 50%, IC_50_ values were obtained. For compound **2**, the most potent BuChE inhibitor, inhibition constants, and mode of inhibition were also calculated. For this purpose, substrate concentrations ranged from ¼ × *K*_M_ to 4 × *K*_M_, and four different inhibitor concentrations were used (0, 10, 25, 50 µM).

### 2.4. Molecular Modelling Studies

The X-ray structures of hMAO-A in complex with harmine (PDB code 2Z5X) [63] and hMAO-B in complex with a coumarin-based inhibitor (PDB code 2V60) [64] were obtained from the Protein Data Bank [65] and used in these studies. Prior to molecular docking, both crystallographic ligand-protein complexes were subjected to an energy minimization aimed at optimizing the orientation of the structural water molecules interacting with the bound inhibitors. The minimization was performed with Amber 16 software (University of California, San Francisco: 2014) [66] using ff14SB force field for the protein and GAFF (General Amber force field) for the ligands. The complexes were located in a rectangular parallelepiped water box and solvated with a 15 Å water shell using the TIP3P explicit solvent model. Sodium ions were added to neutralize the system. A 5000-steps minimization, including of 2000 steps of steepest descent (SD) followed by 3000 steps of conjugate gradient (CG), was carried out, applying a position restraint of 100 kcal/mol·Å^2^ on all receptor and ligand heavy atoms, in order to uniquely minimize the positions of the water molecules and the orientation of rotatable polar hydrogens. The energy minimized receptors, including the relevant structural water molecules, were then used for the docking studies. All nine compounds of the series were initially built using MolBook UNIPI 1.3 software [67], which was also employed to verify that none of the synthesized ligands presented structural moieties associated to pan-assay interference compounds (PAINS) [68,69]. Compounds **7** and **9** were then subjected to docking calculations with GOLD 5.1 software [70], using ChemScore fitness function. The region important for the docking calculations comprehended all residues within 10 Å from the ligand. The coumarins were subjected to 100 genetic algorithm runs, in which the “allow early termination” option was switched off, whereas the possibility for the compound to flip ring corners was switched on, leaving all other settings as their defaults. The root-mean-squared deviation (RMSD) threshold for pose clustering was considered as 2.0 Å. The best docked conformation in the best cluster of solutions was evaluated for each compound in the entire docking analysis. The predicted ligand-protein complexes were then energy minimized with Amber 16 using a two-stage minimization protocol. The complexes were solvated and parametrized as performed for the reference X-ray structures and then subjected to a two-stage minimization consisting of 5000 total steps of SD/CG algorithms. First, the enzyme was kept rigid with a position restraint of 100 kcal/mol·Å^2^, then energy minimized by employing a harmonic potential of 10 kcal/mol·Å^2^ only to the α carbons. Blood–brain barrier permeability predictions were performed using the Online BBB Predictor tool, employing the AdaBoost and SVM algorithms, and using MACCS, Openbabel FP2, Molprint 2D, and PubChem fingerprints for representing the structure of the ligand, for a total of eight different combinations [71].

### 2.5. In Vitro Biological Assays

#### 2.5.1. Cell Cultures

The rat cell line CTX/TNA2 (normal astrocytes) was used to assess the biological activity of compounds **1**–**9** and to evaluate their effectiveness to counteract oxidative stress-induced neuroinflammation. The CTX-TNA2 rat astrocyte cell line was provided by the European Collection of Cell Cultures (ECACC, Merck, Darmstadt, Germany) and maintained in DMEM High glucose supplemented with 10% of FBS and 1% penicillin-streptomycin (100 μg/mL) (EuroCloneSpA Life-Sciences-Division, Milano, Italy) according to the EACC’s instructions. Cells were grown at 37 °C in a humified atmosphere of 5% CO_2_ [72].

#### 2.5.2. Cell Treatments

Cells were seeded (8 × 10^3^/well) and were left to adhere for 24 h in 96-well plates (Falcon^®^, Corning Incorporated, NY, USA). The medium was removed, incubating the cell monolayer in the presence of increasing concentrations of DEP, AAZ, and test compounds (0–150 µM) for 24–72 h. In a second set of experiments, cells were pre-incubated in the presence of 0.1 µg/mL LPS (lipopolysaccharide from *E. coli,* purchased from Merck, Darmstadt, Germany) for 20 h and further exposed to DEP, AAZ, and test compounds in the same experimental conditions.

#### 2.5.3. Cell Metabolic Activity Assay (MTT)

Cell metabolic activity of CTX/TNA2 rat astrocytes was measured by 3-(4,5-dimethylthiazol-2-yl)-2,5-diphenyltetrazolium bromide assay (MTT, Merck, Milan, Italy). After the exposure, cells were incubated with 100 μL/well of MTT (1 mg/mL), diluted 1:10 with fresh growth medium for 4 h at 37 °C and 5% CO_2_. Then, the MTT solution was removed to be replaced with 100 μL/well of DMSO. Cells were further incubated for 20 min in the same conditions and gently swirled for 10 min at r.t.

### 2.6. Hydrogen Peroxide Measurements

At 24 h, cell supernatants were taken from the cultures used for the MTT assay. The quantitative determination of H_2_O_2_ released in supernatants was performed by means of a H_2_O_2_ colorimetric kit (cat. no. ADI-907-015, Enzo Life Sciences, Inc., Farmingdale, NY, USA). Then, 50 µL of supernatant/well was used and 100 µL of color reagent was added in each well. Next, wells were mixed and afterwards incubated for 30 min at r.t. The OD was measured following the manufacturer’s instructions.

### 2.7. IL-6 Secretion by ELISA Assay

The amounts (pg/mL) of interleukin-6 (IL-6) were analyzed in cell supernatants using an ELISA kit (Enzo Life Sciences Inc., Lausen, Switzerland) following the manufacturer’s instructions. Absorbance was read at 450 nm and cytokine concentration was determined [73]. Data obtained were normalized on MTT optical densities.

## 3. Results and Discussion

### 3.1. Design and Synthesis

**Scheme 1:**
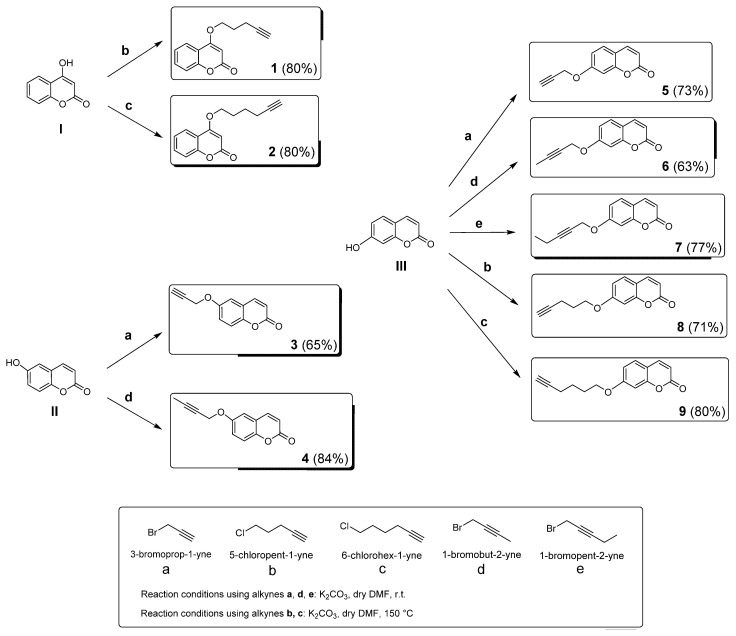
Synthetic procedures for the preparation of coumarin derivatives **1**–**9**. Yield percentages are reported within brackets for each final compound.

The small compound library reported here was designed to explore the chemical space around the coumarin ring that may lead to potent multitarget inhibitors for the treatment of neurodegenerative disorders. Differently substituted coumarin derivatives designed to act as MAO and ChE inhibitors have been widely explored to date. Less attention was given to the intrinsic ability of such scaffold to target another crucial partner in the regulation of oxidative stress and progression of neurodegenerative disorders, the CA enzyme family. Alkyl chains were substituted on the 4, 6, and 7 positions of the ring, making use of alkyl halides bearing both terminal and internal alkyne functions (alkynes **a**–**e**, Scheme 1), the latter being usually less investigated than the former. Position 7, considered as a privileged position when designing potent hMAOs inhibitors [37], was the most explored among the series (derivatives **5**–**9**). On the other hand, position 3, whose derivatization usually leads to potent MAO and ChE inhibitors, was excluded for this study. This choice was based on the weak CA inhibitory activity (falling into the micromolar range) often observed when position 3 of the coumarin ring is substituted [74,75].

Differently substituted alkyl coumarins **1**–**9** were all synthesized by reacting 4-, 6-, and 7-substituted hydroxy coumarins with an alkyl halide, using potassium carbonate (K_2_CO_3_) as a base. Reactions were conducted at room temperature (r.t.) or 150 °C, based on the alkyne substrate (Scheme 1). After purification, the compounds were characterized by TLC (R*f*), ^1^H, and ^13^C NMR spectra, while the purities (>95%) were estimated HPLC analysis. These data are reported in the Methods and Materials and Supplementary Materials.

### 3.2. In Vitro Biological Evaluation: hCA Inhibition

Alkyl coumarins **1**–**9** have been explored as inhibitors of hCAs I, II, VII, IX, and XII through the stopped flow CO_2_ hydrase assay [58]. The inhibition data, in comparison to the standard inhibitor acetazolamide (AAZ), are described in Table 1. The coumarins here investigated are all potent and selective inhibitors of hCA isoforms VII, XI, and XII, while being devoid of inhibitory activity against the two off-targets isoforms hCA I and II. The hCA VII, abundantly expressed in the CNS, was inhibited by compounds **1**–**9** to a similar extent, with *K*_I_ values ranging between 46.93 nM and 77.14 nM. Slight differences must be ascribed to the position and type of substituents substituted on the coumarin scaffold. The hex-1-ynyl group placed at the position 4 (to give compound **2**) and at position 7 (compound **9**) led to the best and the worst hCA VII inhibitors of the series, respectively. Among the 7-substituted derivatives, better results were obtained by reducing the alkyne chain length, with propargylic derivative **5** being the most potent against this isoform. On the other hand, shifting the terminal alkyne to an internal position as with compounds **6** and **7**, did not enhance inhibition to a great extent. The cancer-related isoforms, hCA IX and XII, involved also in inflammatory pathways, were effectively inhibited by most of the coumarins, with nanomolar *K*_I_ values. The only exceptions were derivatives **3** and **5**, the propargyl substituted coumarins, which exhibited a significant reduction in the inhibitory activity regardless of the position of substitution (6 or 7, respectively). Interestingly, the addition of a terminal methyl group to the propargyl alkyne yielded the but-2-ynyl-based derivatives (compounds **4** and **6**, substituted in 6 and 7 positions, respectively), providing a noticeable improvement of inhibitory activity when compared to the *K*_I_ values obtained with the parent propargylic compounds. In a similar manner, the further extension of the chain to obtain the pent-2-ynyl substituted derivatives was favorable for hCA IX and XII inhibition (derivative **7**). A further comparison can be performed between the parent compounds **1** versus **8**, and **2** versus **9**, substituted with the pent-1-ynyl and hex-1-ynyl moieties, respectively. The coumarin ring of compounds **1** and **2** was substituted at the position 4, while analogues **8** and **9** were substituted with the alkyne chain at position 7 (Table 1). From *K*_I_ data, it may be concluded that these substituents were better tolerated at the position 4 than 7, even if only slightly. This evidence is more obvious for hCA IX and hCA XII, which are inhibited at nanomolar concentrations by most of these compounds.

### 3.3. In Vitro Biological Evaluation: hMAO-A and B Inhibition

Compounds **1**–**9** were tested for the capability to inhibit hMAO-A and hMAO-B according to the published protocol [59,60]. IC_50_ values for hMAO-A and hMAO-B, compared to those of curcumin, used as reference drug, are reported in Table 2. The selectivity index (SI), evaluated as the ratio IC_50_ hMAO-A/IC_50_ hMAO-B, is also reported to express the specificity of inhibition of one isoform over the other. An SI>1 defines compounds that preferentially act against hMAO-B, while SI < 1 indicates that a compound preferentially inhibits hMAO-A. Finally, SI ~ 1 identifies compounds with similar IC_50_ against the two isoforms. Data analysis shows that 4-substituted compounds **1** and **2** and 6-substituted compounds **3** and **4** were weak and less efficient inhibitors of both hMAO isoforms when compared to the reference drug, curcumin, with IC_50_ values higher than 10 µM. In particular, compounds **1** and **2**, bearing on the 4 position of the coumarin ring the pent-1-yne and hex-1-yne substituents, respectively, were found to be selective hMAO-A inhibitors, although with micromolar IC_50_ values. A similar inhibitory profile was observed for the 6-substituted compound **3**, bearing a propargylic substituent, whereas the addition of a methyl group to the propargyl moiety to give compound **4** increased the inhibitory potency against hMAO-B, resulting in selective hMAO-B inhibition. Overall, the weak inhibitory activity of derivatives **1**–**4** confirmed that substitution of the coumarin ring at positions 4 and 6 are less advantageous compared to positions 3 and 7, as previously reported [37]. With the exception of the propargyl moiety (compound **5**), the same substituents placed at the position 7 (compounds **5**–**9**) afforded sub-micromolar/nanomolar inhibitors. Moreover, compounds **6**–**9** were found to be more potent hMAO-B inhibitors than the reference drug curcumin. As for hMAO-A, comparable or lower IC_50_ values when compared to curcumin were observed only for compounds **8** and **9**, respectively.

Within the 7-substituted series, increasing of substituent chain length and flexibility elicited an increase in hMAO-A inhibition, with the 7-substituted hex-1-ynyl derivative (compound **9**) being the most potent hMAO-A inhibitor of the series. Compound **9** was also a potent hMAO-B inhibitor, with low nanomolar activity against this isoform (IC_50_ = 0.007 μM); similar inhibitory activity was also observed for derivative **7**. However, the two compounds considerably differed in their selectivities towards the two hMAO isoforms. In fact, analogue **7** was the most selective hMAO-B inhibitor with SI = 1396; on the contrary, compound **9** displayed a reduced selectivity index (SI = 92.8). This difference may not be ascribed to differences in hMAO-B inhibitory activity since the two compounds possess similar IC_50_ values (Table 2). The difference in SI was attributed to different binding of the substituents to the active sites of the hMAOs. In this respect, reduced flexibility of the pent-2-ynyl moiety leads to weaker hMAO-A inhibition (see molecular modeling studies). As for derivatives **6** and **8**, similar IC_50_ values were observed for the inhibition of hMAO-B, both being in the high nanomolar range, with compound **6** being slightly more potent than compound **8**. Conversely, an increase in the chain length and flexibility improved the inhibitory activity against hMAO-A, leading to a concomitant reduction in SI value of **8** compared to compound **6**.

Interestingly, when comparing compound **7** with compound **8**, both possessing a substituent of the same length but differing in the position of the alkyne within the chain, it is evident that the terminal alkyne present in compound **8** results in weaker hMAO-B inhibition, while improving the hMAO-A inhibitory potency. However, considering that further elongation of the alkyne chain (compare **8** versus **9**) restored the inhibitory activity against the B isoform, it seems reasonable to conclude the following for position 7: (i) small structural modifications have a large impact on the compound’s ability to efficiently inhibit hMAO-B, whereas a less significant effect is observed for hMAO-A with all the compounds inhibiting in the low-medium micromolar range; (ii) chain length and, to a greater extent, chain flexibility are crucial factors in influencing hMAO-A inhibitory potencies; conversely, this trend is not observed for hMAO-B where precise structural determinants need to be present in order to induce the proper compound orientation and interactions within the active site.

It is important to highlight that compounds **7** and **9** act as nanomolar inhibitors of the biological targets considered so far in this study, hMAO-B and hCA isoforms VII, IX, and XII, involved in neurodegeneration process and neuroinflammation. This is an important aspect when the objective is to obtain a balanced multi-target directed ligand.

**Figure 2:**
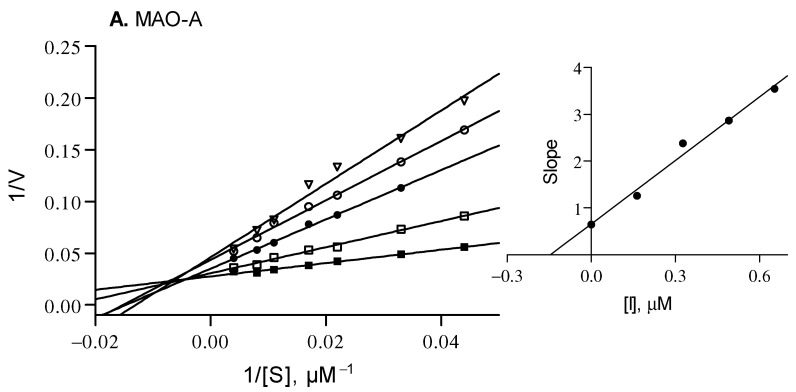

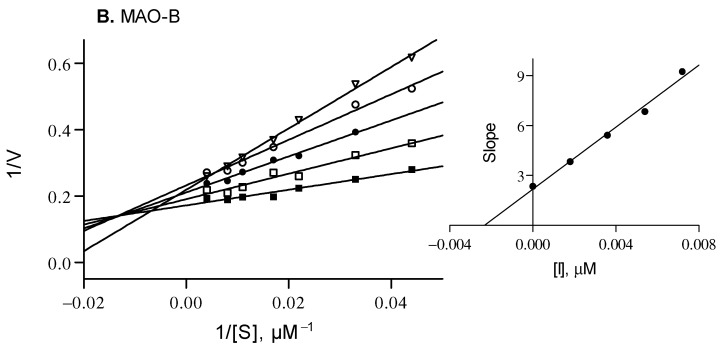
Lineweaver–Burk plots for the inhibition of hMAO-A and hMAO-B by compound **9**. The following inhibitor concentrations were used for the Lineweaver–Burk plots: 0 × IC_50_ (filled squares), ¼ × IC_50_ (open squares), ½ × IC_50_ (filled circles), ¾ × IC_50_ (open circles) and 1 × IC_50_ (triangles). The subfigures are graphs of the slopes of the Lineweaver–Burk plots as a function of different inhibitor concentrations. *K*_i_ values of 0.146 and 0.0023 µM for MAO-A and MAO-B were calculated, respectively.

Compound **9** was selected as a representative inhibitor to further explore, through enzymatic kinetic studies, the hMAO inhibition mechanism. Lineweaver–Burk plots have been obtained for hMAO-A and hMAO-B, and compound **9** was found to exhibit *K*_i_ values of 0.146 µM and 0.0023 µM for the inhibition of hMAO-A and hMAO-B, respectively (Figure 2). The Lineweaver–Burk plots were linear and intersected close to the y-axis while graphs of the slopes as a function of different inhibitor concentrations were also linear. This suggests a competitive inhibition mechanism, and thus reversible interaction with the enzyme. It is reasonable to assume that this inhibition mechanism is shared by all the compounds here reported.

### 3.4. In Vitro Biological Evaluation: AChE and BuChE Inhibition

Along with the ability to effectively bind to the hMAO-B active site, the planar ring system of coumarin scaffold also allows the interaction with the peripheral anionic site (PAS) of AChE. Therefore, it is not surprisingly that numerous multi-target directed ligands are based on this scaffold [11]. With the aim to evaluate their ability to regulate CNS acetylcholine levels, compounds **1**–**9** were evaluated as putative AChE (from *Electrophorus electricus*, electric eel) and BuChE (from equine serum) inhibitors, using the well-known Ellman’s colorimetric assay [76]. Such enzymes share a high structural similarity with the human isoforms [77]. The obtained IC_50_ values, compared to those of galantamine, used as reference drug [62], are disclosed in Table 3. Although most of the derivatives were ineffective against AChE, the most promising hMAO-B inhibitors, **7** and **9**, showed 50% inhibition of this enzyme at 100 µM concentration, and were the only active compounds of the series. The same derivatives weakly inhibited also BuChE, with IC_50_ values in the high micromolar range. Coumarins **1** and **2**, bearing substituents at position 4 of the coumarin ring, displayed the most potent inhibition of BuChE, albeit in the micromolar range. For compound **2**, the most potent BuChE inhibitor, graphical analysis of the Cornish–Bowden plots relative to the eqBuChE activity yielded both *K*_ia_ and *K*_ib_ values. These kinetic studies allowed to elucidate the inhibition mode of compound **2**, revealing a mixed inhibition mechanism.

### 3.5. Molecular Modeling Studies

**Figure 3:**
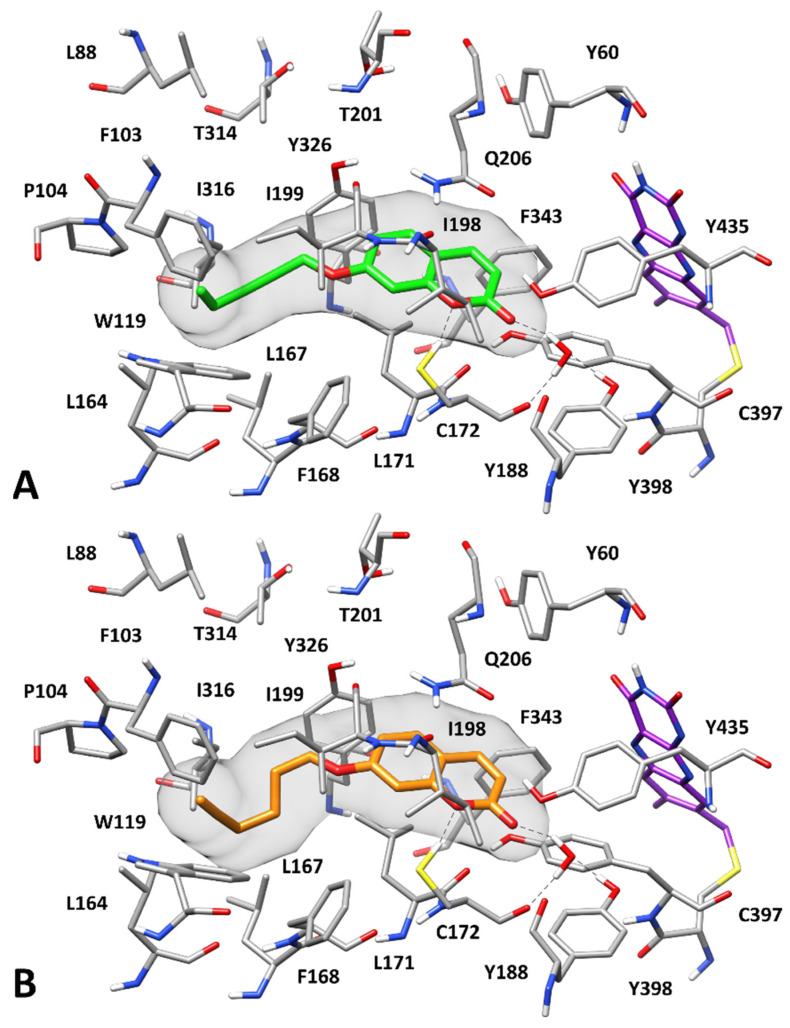
Predicted binding modes of **7** (**A**) and **9** (**B**) to hMAO-B. Compounds **7** and **9** are shown in green and orange, respectively. The flavin group of the cofactor is shown in purple. The molecular surface of the ligands is shown in gray. H-bonds are depicted as black dashed lines.

Molecular docking refined by energy minimization in explicit solvent of the predicted ligand-protein complexes were employed in order to rationalize the inhibitory activities against the hMAOs, as well as the selectivity for hMAO-B over hMAO-A, which were experimentally observed for the synthesized coumarin derivatives. Compounds **7** and **9**, which showed the best activity and selectivity profile, were used in these studies. Initially, the computational protocol was applied to predict the potential binding modes of the two ligands to the catalytic site of hMAO-B, since these compounds potently inhibited hMAO-B with low nanomolar potencies and displayed very similar activities (with IC_50_ values of 8 nM and 7 nM for **7** and **9**, respectively). As shown in Figure 3, the two ligands were predicted to have a comparable binding mode, in which the coumarin core of both inhibitors is placed in the inner portion of hMAO-B binding site, in proximity to the flavin moiety of the cofactor, forming direct and water-bridged H-bonds that anchor the ligands to the enzyme.

In particular, the endocyclic oxygen of the coumarin core forms an H-bond with the sulfhydryl group of C172, while the carbonyl group of the inhibitors establishes an H-bond network with the hydroxyl group of Y188 and the backbone oxygen of C172 mediated by a structural water molecule. Moreover, the bicyclic scaffold of the two inhibitors forms π–π interactions primarily with Q206, Y326, and Y398 and shows lipophilic interactions with L171, I198, and I199. Finally, the alkynyl substituents of the compounds are placed in a hydrophobic pocket defined by F103, P104, W119, L164, L167, F168, I199, and I316, thus forming both lipophilic and aromatic interactions with these residues.

The same computational protocol was then employed for predicting the putative binding orientations of the two ligands to the hMAO-A catalytic site, in the attempt of rationalizing their selectivity for hMAO-B over hMAO-A.

**Figure 4:**
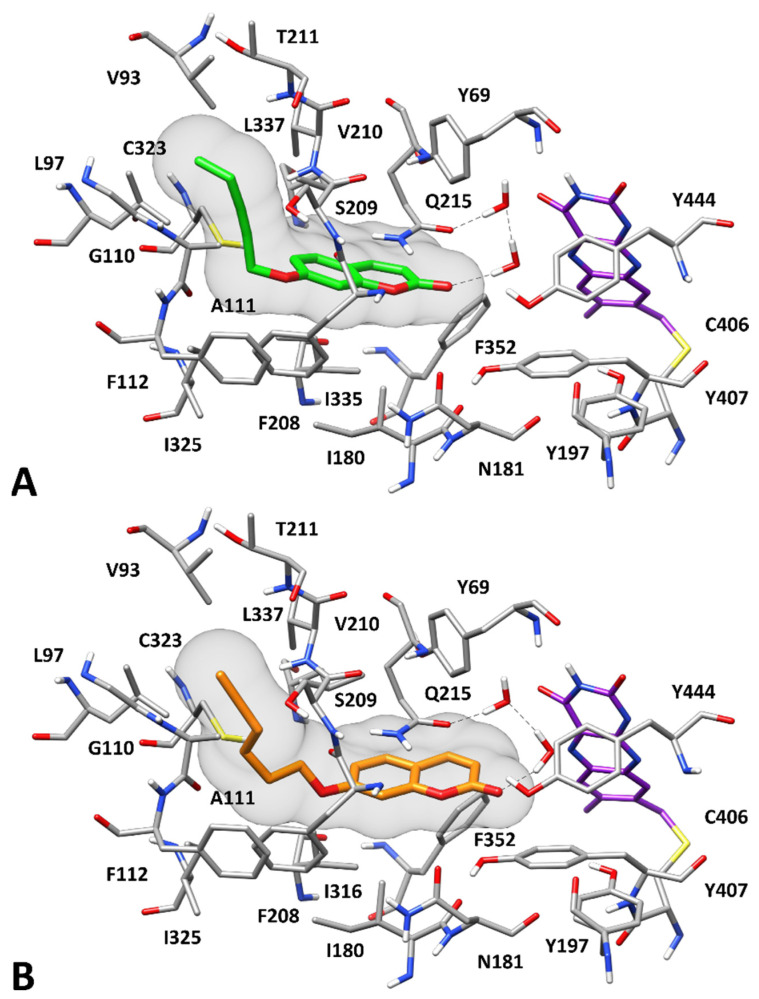
Predicted binding modes of **7** (**A**) and **9** (**B**) to hMAO-A. Compounds **7** and **9** are shown in green and orange, respectively. The flavin group of the cofactor is shown in purple. The molecular surface of the ligands is depicted in gray. H-bonds are shown as black dashed lines.

Figure 4A shows that compound **7** binds to hMAO-A quite differently compared to that predicted for hMAO-B. This appears to be due to the presence of some non-conserved residues among the two enzyme isoforms that determines a different shape of the binding pocket and a different pattern of receptor anchoring sites. The side chain of F208 of hMAO-A (the analogous residue to I199 of hMAO-B) prevents binding to the hydrophobic pocket that, in hMAO-B, was occupied by the alkynyl substituent of the two ligands. On the contrary, a lateral lipophilic channel which is linked to the solvent and defined by V93, L97, G110, A111, F208, S209, and V210, is open in hMAO-A due to the presence of I335 instead of the bulky side chain of Y326 in hMAO-B. Since this channel is located on the side with respect to the inner part of the binding site where the flavin moiety of the cofactor is placed, the catalytic pocket of hMAO-A is more L-shaped than linear, compared to that of hMAO-B. For this reason, compound **7** cannot be properly accommodated in the hMAO-A binding site. The rather rigid substituent of the ligand fits well within the lateral lipophilic channel, forming hydrophobic interactions with V93, L97, A111, F208, and V210; however, its poor flexibility prevents the coumarin moiety from protruding into the inner portion of binding pocket. Although the compound shows favorable interactions with Q215 and I335, it lacks the strong π–π interactions with the aromatic amino acids in the region of the cofactor. Moreover, since the carbonyl group of the ligand is distant from Y197 (the analogous residue to Y188 of hMAO-B) and the C172 of hMAO-B is replaced by N181 in hMAO-A, the compound can only form a weak interaction with the side chain of Q215 mediated by two water molecules. Taken together, these considerations may explain the reduced inhibition potency of **7** for hMAO-A (with an IC_50_ of 11.2 μM) compared to that observed for hMAO-B, and thus its selectivity for hMAO-B. In contrast, compound **9** maintained a submicromolar inhibitory activity against hMAO-A (with an IC_50_ of 0.65 μM), hence showing a less marked selectivity for hMAO-B. The binding mode predicted for this ligand in hMAO-A (Figure 4B) suggests that the more flexible tail of the molecule allows the inhibitor to better fit into the enzyme catalytic pocket, thus binding in proximity of the cofactor and interacting with Y69, F352, and Y407, while preserving all other ligand-protein interactions predicted for compound **7**. This may explain the different activity and selectivity profile of compound **9** compared to compound **7**. Finally, with the aim of evaluating the suitability of the two ligands as potential CNS drug leads, in silico predictions of their blood–brain barrier (BBB) permeability were performed using the Online BBB Predictor tool, which is specifically designed for BBB-permeability predictions [71]. The results suggested that compounds **7** and **9** are able to cross the BBB, and should be thus suitable as CNS therapeutics, since they were both labelled as BBB-positive by all eight predictive methods available in the web tool (see Methods and Materials for details).

### 3.6. In Vitro Biological Evaluation: Cellular Assays

**Figure 5:**
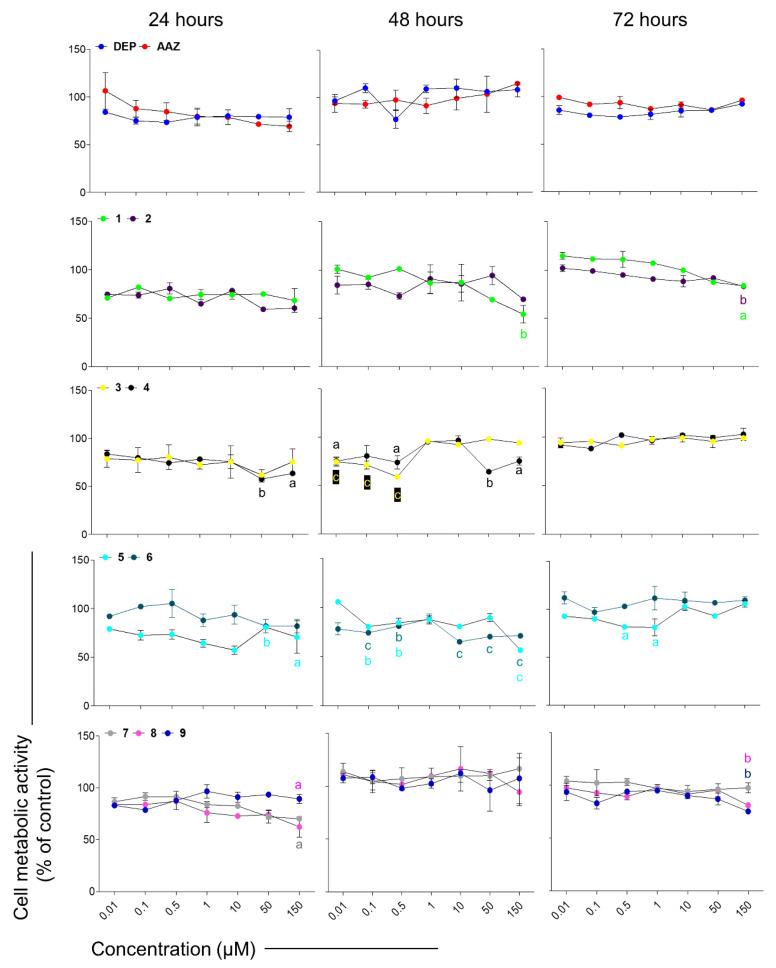
Cell metabolic activity of rat astrocytes exposed to deprenyl (DEP), AAZ and test compounds for 24, 48, and 72 h. Trend lines represent cell metabolic activity percentages. The untreated control (not shown) was considered as 100%. Data are the means ± standard deviations obtained from three independent experiments in triplicates (n = 9). a = *p* < 0.01, b = *p* < 0.001 and c = *p* < 0.0001 comparing treated cells and the untreated control.

First, to assess the biocompatibility of compounds, the MTT test was performed under non-inflammatory conditions up to 72 h of treatment. Deprenyl and AAZ were used as reference compounds (Figure 5). Compounds **1** and **2** show a good biocompatibility, with a cell metabolic activity significantly decreased only after 48 h and at the highest concentration tested (150 µM). Compounds **3** and **4** are well tolerated up to 48 h and display a good biocompatibility at the longest exposure time (72 h), showing cell metabolic activity percentages comparable to the one of untreated cells (100%, not shown). In the presence of **5** and **6**, cell metabolic activity significantly decreases at all the concentrations tested at 48 h. After 72 h of treatment, cells counteract the negative effect on their metabolism induced by **5** (only higher concentrations) and **6**. Finally, compounds **7**–**9** are well tolerated by astrocytes, and only displayed an effect on cell metabolic activity at 150 µM.

**Figure 6:**
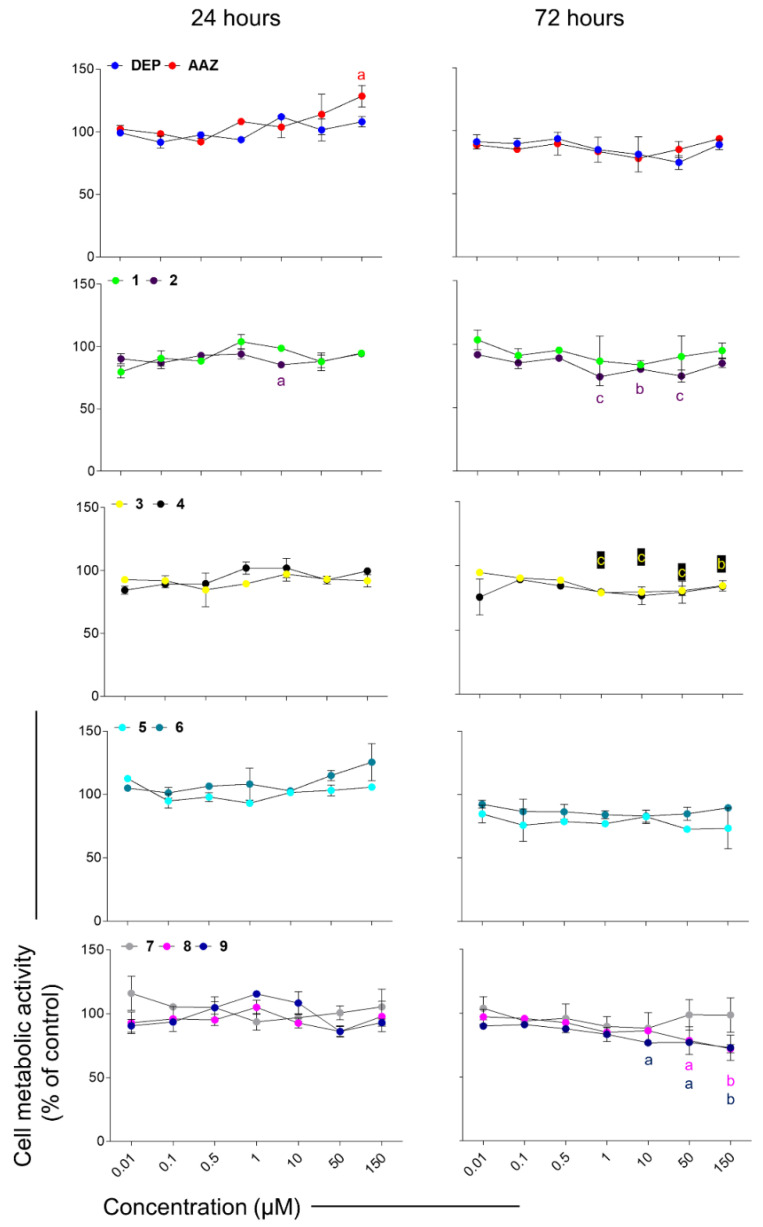
Cell metabolic activity of rat astrocytes pre-stimulated with LPS and exposed to DEP (deprenyl as hMAO-B inhibitor), AAZ (acetazolamide, as hCA inhibitor) and the test compounds for 24 and 72 h. Trend lines represent cell metabolic activity percentages. Cells pre-stimulated with LPS and afterwards exposed to growth medium (not shown) were considered as 100%. Values are the means ± standard deviations obtained from three independent experiments in triplicates (n = 9). a = *p* < 0.01, b = *p* < 0.001 and c = *p* < 0.0001 between cells pre-stimulated with LPS and afterwards treated with coumarins and cultures pre-stimulated with LPS and afterwards exposed to growth medium (control).

To establish oxidative stress-related neuroinflammation, rat astrocytes were pre-stimulated with a subtoxic concentration of LPS, which can induce inflammation without being cytotoxic. It has been widely demonstrated that astrocytes react to nutrients and hormones and can be activated by bacterial products such as LPS, leading to secretion of pro-inflammatory mediators and cytokines [78]. Moreover, astrocytes are energy regulatory centers in the CNS and glycogen, the major representative of energy storage in the brain, is present almost exclusively in astrocytes and is typically consumed through aerobic glycolysis [79]. Moreover, astrocytes switch from the ‘resting’ to the ‘reactive’ state, experiencing a metabolic adaptation to meet their energy demands in response to injury or stress or inflammation [80]. After being pre-stimulated with LPS, CTX cells slightly but significantly increase their metabolic activity. To simplify the visualization of the results, MTT data are therefore here normalized with respect to optical densities obtained from cells pre-stimulated with LPS only (control sample set as 100%) (Figure 6). Subsequently, compounds able to counteract the increase in cell metabolic activity of astrocytes might be suitable candidates as anti-inflammatory compounds. In this light, compounds **2**, **3**, **8**, and **9** seem able to counteract the LPS-induced increase in cell metabolic activity, since these compounds display percentages significantly lower than the one of control (LPS only).

**Figure 7:**
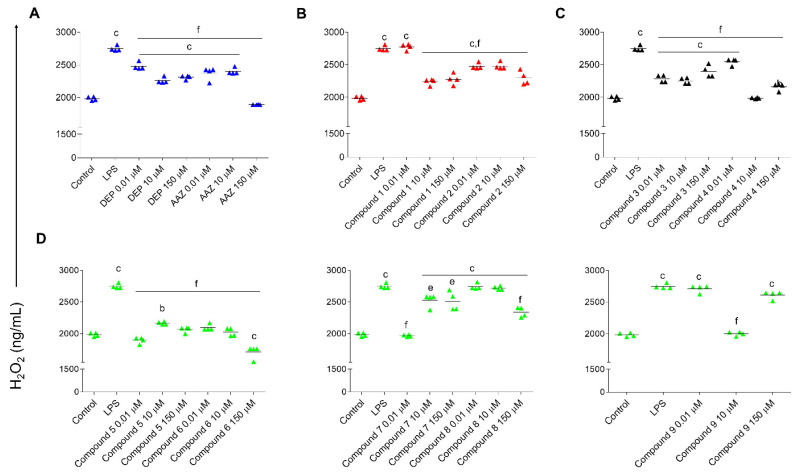
H_2_O_2_ generation in rat astrocytes pre-stimulated with LPS and exposed to DEP, AAZ, and test compounds for 24 h. Graphs show the amount of H_2_O_2_ measured as ng/mL related to AAZ and DEP (**A**), **1** and **2** (**B**), **3** and **4** (**C**) and **5**, **6**, **7**, **8**, and **9** (**D**). Single replicates are represented by colored triangles and values are the means ± standard deviations of two independent experiments in duplicates (n = 4). Values are normalized with respect to the optical densities obtained from the MTT assay. Control: untreated cells (exposed to growth medium for the entire experimental procedure). LPS: cells pre-stimulated with LPS and afterwards exposed to growth medium. b = *p* < 0.001 and c = *p* < 0.0001 between cells pre-stimulated with LPS and afterwards treated with coumarins and cultures pre-stimulated with LPS and afterwards exposed to growth medium (control); e = *p* < 0.001 and f = *p* < 0.0001 between and cells pre-stimulated with LPS and afterwards treated with coumarins and cultures pre-stimulated with LPS and afterwards exposed to growth medium.

The CNS is characterized by a high metabolism, leading to the production of harmful amounts of free radicals, and it can be extremely vulnerable to oxidative stress [81,82]. It has been reported that in a mouse model of AD, Aβ plaques activate MAO-B, thus increasing oxidative stress through the production of H_2_O_2_. Excessive amounts of H_2_O_2_ can potentially affect astrocytes both morphologically and epigenetically, as described for other cells [83]. In another work, it has been demonstrated that toxin-triggered H_2_O_2_ generation via catabolic pathways in astrocytes can induce serious and permanent astrocyte responsiveness and consequent neurodegeneration [84]. In our experimental model, the stimulation with LPS induces a significant increase in the H_2_O_2_ generation (2749 ng/mL) compared to untreated astrocytes (1986 ng/mL) (Figure 7). Notably, all the tested compounds decreased the production of H_2_O_2_. The most effective molecules able to counteract the LPS-induced increase in H_2_O_2_, restoring it to the untreated control levels, are **5** and **7** at 0.01 µM, **4** and **9** at 10 µM, and **6** at 150 µM, disclosing **5** and **7** as the most promising compounds.

**Figure 8:**
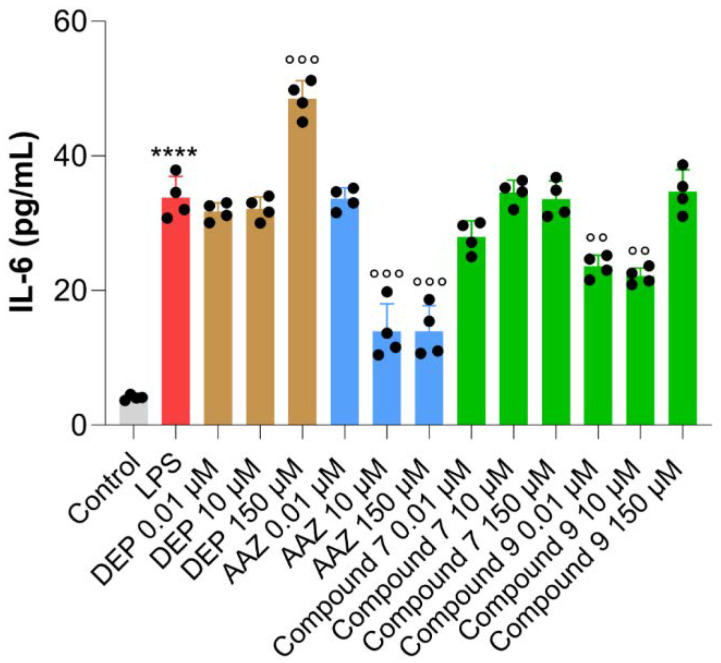
Interleukin-6 secretion from rat astrocytes pre-stimulated with LPS and exposed to DEP, AAZ, and test compounds **7** and **9** for 24 h. Black circles represent single replicates and values are the means ± standard deviations of two independent experiments in duplicates (n = 4). Values are normalized with respect to the optical densities obtained from the MTT assay. Control = untreated cells (cells exposed to growth medium for the entire experimental procedure). LPS = cells pre-stimulated with LPS and afterwards exposed to growth medium. **** *p* < 0.0003 comparing cells pre-stimulated with LPS and untreated cells. °° *p* < 0.001 and °°° *p* < 0.0001 among cells pre-stimulated with LPS and cells pre-stimulated with LPS and afterwards exposed to tested coumarins.

Various cell types (neurons, microglia, astrocytes, epithelial cells, and macrophages) can produce Interleukin (IL)-6 in the CNS. Research studies have demonstrated that Toll-like receptor (TLR)4 stimulation in astrocytes by LPS increases the secretion of the pro-inflammatory cytokine IL-6 [85,86], leading to neuroinflammation and consequent neurodegeneration [87]. Against this background, IL-6 secretion was analyzed in LPS-stimulated rat astrocytes exposed to DEP, AAZ and the most effective test compounds **7** and **9** (Figure 8). As expected, the highest concentrations of AAZ are capable of decreasing IL-6 secretion in a significant manner compared to LPS alone, as already reported [88]. On the other hand, DEP increases the amount of IL-6 at the highest concentration tested as observed elsewhere [89,90]. In parallel, **7** is not effective on the modulation of IL-6 production from LPS-stimulated astrocytes, whereas **9** decreases the amount of the cytokine at 0.01 and 10 µM. Collectively, compounds **9** and **7** share the same inhibitory profile against hMAO-B, human CAs, and cholinesterases. Conversely, compound **9** is more potent against hMAO-A, thus displaying an inferior isoform selectivity. It is possible that this balanced inhibition could be responsible for such slight biological differences (compound **7** gave a strong reduction in H_2_O_2_ release at 0.01 μM and at the same concentration slightly decreased IL-6 production). Compound **5** had an effect on IL-6 release, but it is likely mediated by other mechanisms or antioxidant targets. This is clear from the analysis of the inhibitory data. With respect to compounds **7** and **9**, compound **5** was less active against hCA IX and XII, less active against hMAO-B (also less active than compound **9** against MAO-A), and less active against cholinesterases. Thus, it is possible to speculate that only compounds **7** and **9** can have a modulatory effect against inflammation directly linked to enzyme inhibition.

## 4. Conclusions

In summary, in the present work, a multitarget approach to tackle neuroinflammation has been explored. In this study, 4-, 6-, and 7-alkyl substituted coumarins have been synthesized and assayed as BBB permeant inhibitors of many enzymes involved in neurodegeneration (CAs, MAOs, and ChEs). All the coumarins were effective and selective inhibitors of the human CA isoforms of interest (hCA VII, IX, and XII), over the off-targets isoforms hCA I and II. 4-Substitution on the coumarin ring (compounds **1** and **2**) was particularly favorable for BuChE inhibition, although with high *K*_I_ values observed. 6-Substituted coumarins **3** and **4** were found to act as potent and selective hCA IX and XII inhibitors, weak hMAO A and B inhibitors (IC_50_ values within the high micromolar range), and completely ineffective against AChE and BuChE enzymes. As for 7-substituted compounds (compounds **5–9**), different activities were observed, with compounds **5**, **6**, and **8** acting as dual hMAO-B/hCA inhibitors, whereas compounds **7** and **9** displayed also weak AChE and BuChE inhibitory activities.

Interestingly, in vitro biological evaluation on rat astrocytes confirmed the importance of the multitarget approach. The results indicated compounds **2**, **3**, **8**, and **9** as the most promising in counteracting the increase in cellular metabolic activity induced by LPS. On the other hand, compounds **5** and **7** were found to be to be very effective in decreasing LPS-induced H_2_O_2_ generation, and **9** was found effective in decreasing IL-6 amounts, thus possessing potential neuroprotective activities. In this light, compounds **7** and **9** can be considered as the most promising among the series, being able to effectively inhibit multiple cellular targets involved in neurodegeneration (CAs, MAOs, AChE, and BuChE) and explicating neuroprotective activities in LPS-prestimulated astrocytes, counteracting the oxidative stress-induced neuroinflammation. The main limitations of this study could be the suitability of these compounds as CNS drugs in vivo, but several technological formulations were also previously suggested for the coumarin scaffold [91,92,93]. In addition, the established in vitro inflammatory experimental model could be optimized for a different LPS stimulation and analysis of additional time points, trying to mimic the proinflammatory environment in the CNS, where ILs and chemokines are continuously produced.

## Figures and Tables

**Table 1:** Enzymatic inhibition of hCA I, hCA II, hCA VII, hCA IX, and hCA XII with compounds **1**–**9** and the reference sulfonamide inhibitor, acetazolamide (**AAZ**). * Mean of 3 different assays (errors were within ±5–10% of the values).

			*K*_I_ (nM) *				
Entry	Structure	R_1_	*h*CA I	*h*CA II	*h*CA VII	*h*CA IX	*h*CA XII
	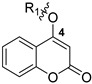						
**1**		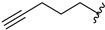	6262.0	>10,000	59.81	17.23	4.8
**2**		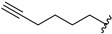	>10,000	>10,000	46.93	9.0	4.8
	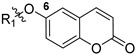						
**3**		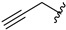	>10,000	>10,000	61.72	4747.6	56.4
**4**		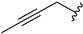	>10,000	>10,000	63.27	24.2	4.3
	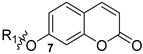						
**5**		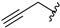	>10,000	>10,000	51.87	1350.0	730.0
**6**		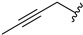	>10,000	>10,000	68.17	9.3	6.8
**7**		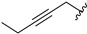	>10,000	>10,000	63.48	13.0	6.8
**8**		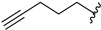	>10,000	>10,000	69.3	26.6	6.5
**9**		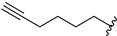	>10,000	>10,000	77.14	31.3	5.9
**AAZ**	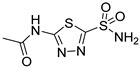		250	12.1	2.5	25.8	5.7

**Table 2:** IC_50_ values for hMAO-A and hMAO-B inhibition by compounds **1**–**9** and the reference inhibitor, curcumin. * The values are given as the mean ± SD of triplicate determinations. ** Selectivity index for hMAO-B over hMAO-A evaluated as IC_50_ hMAO-A/IC_50_ hMAO-B.

			IC_50_ (μM) *		
Entry	Structure	R_1_	hMAO-A	hMAO-B	SI **
	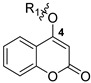				
**1**		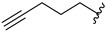	51.2 ± 2.16	>100	<0.51
**2**		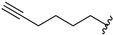	10.8 ± 0.76	25.9 ± 2.54	0.42
	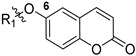				
**3**		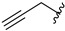	40.2 ± 4.95	65.4 ± 1.21	0.61
**4**		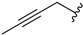	55.7 ± 3.71	12.1 ± 1.33	4.6
	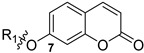				
**5**		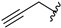	11. 6 ± 0.29	5.79 ± 0.26	1.99
**6**		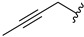	21.3 ± 0.77	0.25 ± 0.017	85.3
**7**		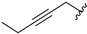	11.2 ± 0.39	0.008 ± 0.00062	1396
**8**		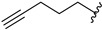	5.70 ± 0.21	0.51 ± 0.0099	11.2
**9**		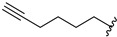	0.65 ± 0.027	0.007 ± 0.00035	92.8
**curcumin**	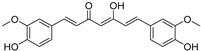		5.54 ± 0.36	4.00 ± 0.17	1.4

**Table 3:** IC_50_ values for the inhibition of AChE and BuChE by compounds **1**–**9**. * The values are given as the mean ± SD of triplicate determinations; *K*_ia_ refers to the inhibition constant for the inhibitor binding the free enzyme. *K*_ib_ is related to the inhibition constant for the inhibitor binding the ES complex.

			IC_50_ (µM) *	
Entry	Structure	R_1_	AChE	BuChE
	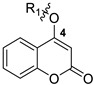			
**1**		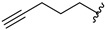	>100	33 ± 1
**2**		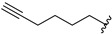	>100	21 ± 1 µM mixed inhibition; *K*_ia_ = 13 ± 3 µM; *K*_ib_ = 25 ± 3 µM
	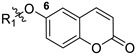			
**3**		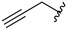	>100	>100
**4**		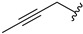	>100	>100
	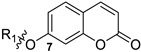			
**5**		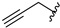	>100	>100
**6**		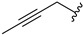	>100	>100
**7**		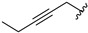	~50% inhibition at 100 µM	59 ± 1
**8**		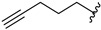	>100	>100
**9**		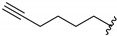	~50% inhibition at 100 µM	51 ± 4
**Galantamine**	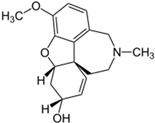		1.29 ± 0.14	5.47 ± 0.40

## Data Availability

Data is contained within the article or supplementary material.

## Supplementary Materials

The following supporting information can be downloaded at: https://www.mdpi.com/article/10.3390/antiox12122044/s1, Supporting Information: HPLC chromatograms for purity determination, ^1^H and ^13^C NMR spectra.
