# Supplementary material for: A Multitarget Approach against Neuroinflammation: Alkyl Substituted Coumarins as Inhibitors of Enzymes Involved in Neurodegeneration
Source: Antioxidants (Basel). 2023 Nov 25;12(12):2044. doi: 10.3390/antiox12122044 (PMC10740956; doi:10.3390/antiox12122044)

Supplementary Materials

A multitarget approach against neuroinflammation: alkyl substituted coumarins as inhibitors of enzymes involved in neurodegeneration

Emanuela Berrino [1,2], Simone Carradori [3,*], Fabrizio Carta [2], Francesco Melfi [3], Marialucia Gallorini [3], Giulio Poli [4], Tiziano Tuccinardi [4], José G. Fernández-Bolaños [5], Óscar López [5], Jacobus P. Petzer [6], Anél Petzer [6], Paolo Guglielmi [1], Daniela Secci [1] and Claudiu T. Supuran [2]

[1] Department of Drug Chemistry and Technologies, Sapienza University of Rome, P.le A. Moro 5, 00185 Rome, Italy; emanuela.berrino@uniroma1.it (E.B.); paolo.guglielmi@uniroma1.it (P.G.); daniela.secci@uniroma1.it (D.S.)
[2] NEUROFARBA Department, Sezione di Scienze Farmaceutiche e Nutraceutiche, Università degli Studi di Firenze, Via Ugo Schiff 6, 50019 Florence, Italy; fabrizio.carta@unifi.it (F.C.);
claudiu.supuran@unifi.it (C.T.S.)
[3] Department of Pharmacy, ''G. d'Annunzio'' University of Chieti-Pescara, via dei Vestini 31, 66100 Chieti, Italy; francesco.melfi@unich.it (F.M.); marialucia.gallorini@unich.it (M.G.)
[4] Department of Pharmacy, University of Pisa, Via Bonanno 6, 56126 Pisa, Italy; giulio.poli@unipi.it (G.P.); tiziano.tuccinardi@unipi.it (T.T.)
[5] Departamento de Química Orgánica, Facultad de Química, Universidad de Sevilla, Apartado 1203,
41012Seville, Spain; bolanos@us.es (J.G.F.-B.); osc-lopez@us.es (Ó.L.)
[6] Pharmaceutical Chemistry, School of Pharmacy and Centre of Excellence for Pharmaceutical Sciences, North-West University, Potchefstroom 2531, South Africa; jacques.petzer@nwu.ac.za (J.P.P.); 12264954@nwu.ac.za (A.P.)
* Correspondence: simone.carradori@unich.it

Table of Contents

HPLC chromatograms for purity determination…………………………………………………..**2**

^{1}H and ^{13}C NMR spectra for new compounds……………………………………………………..**7**

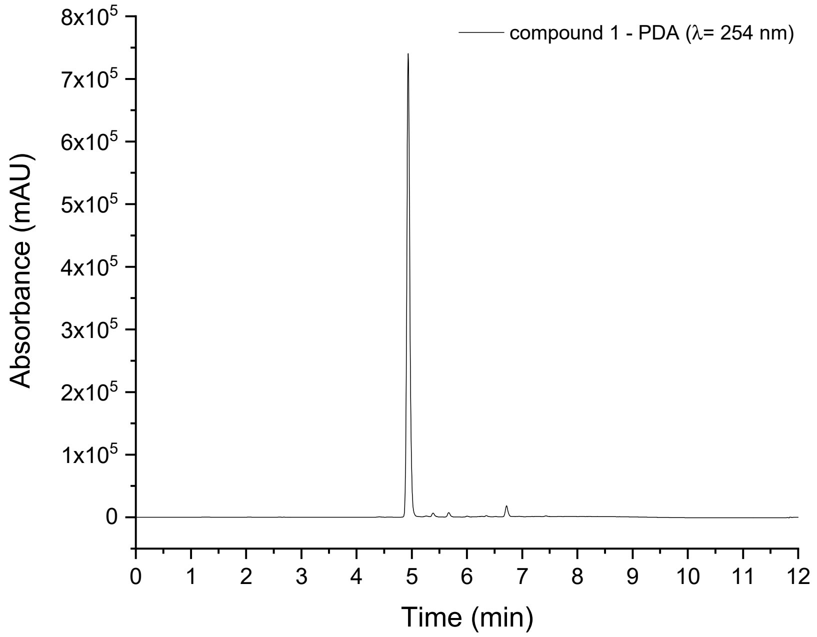

Figures S1. HPLC chromatogram for purity determination of compound **1**.

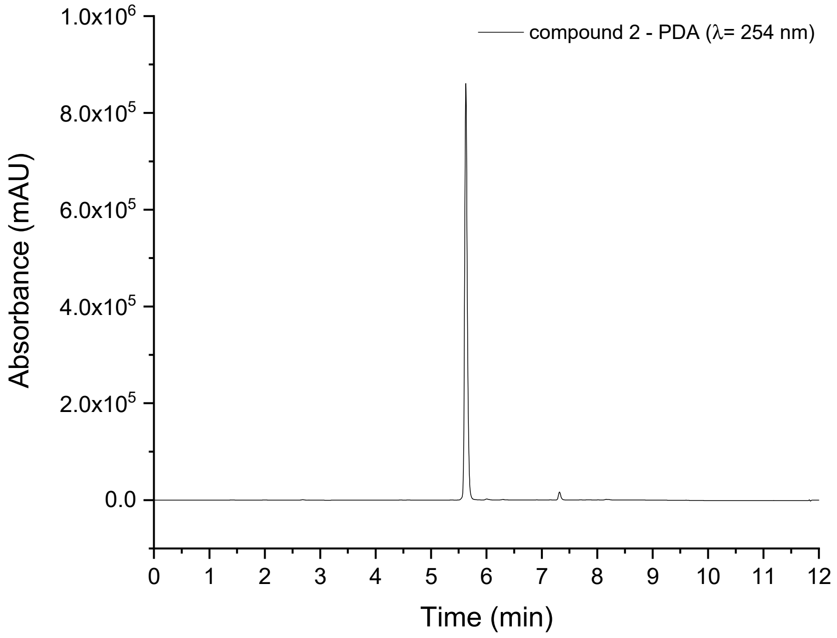

Figures S2. HPLC chromatogram for purity determination of compound **2**.

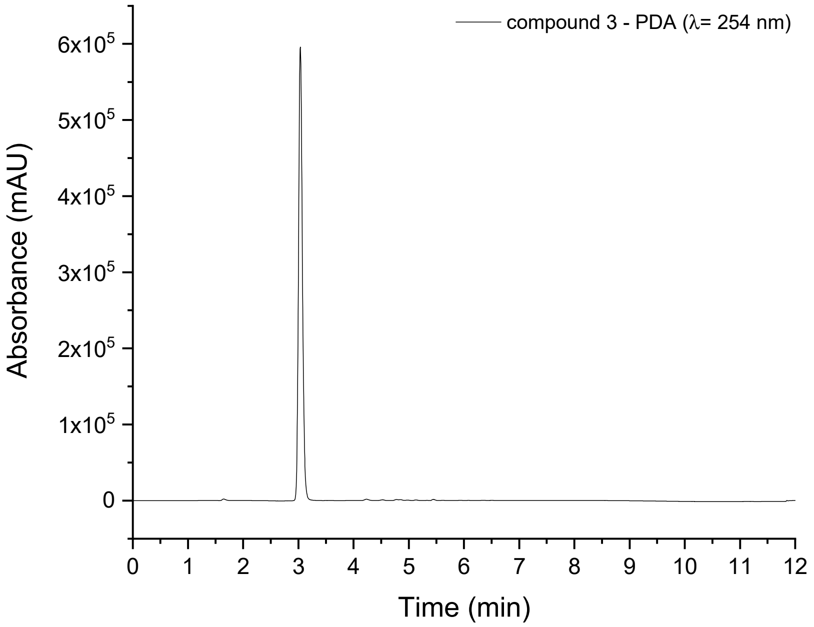

Figures S3. HPLC chromatogram for purity determination of compound **3**.

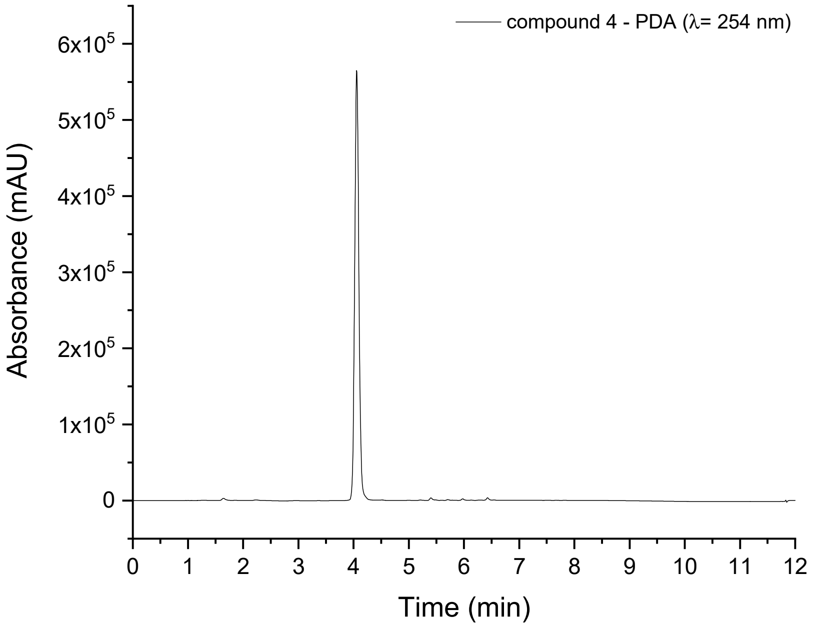

Figures S4. HPLC chromatogram for purity determination of compound **4**.

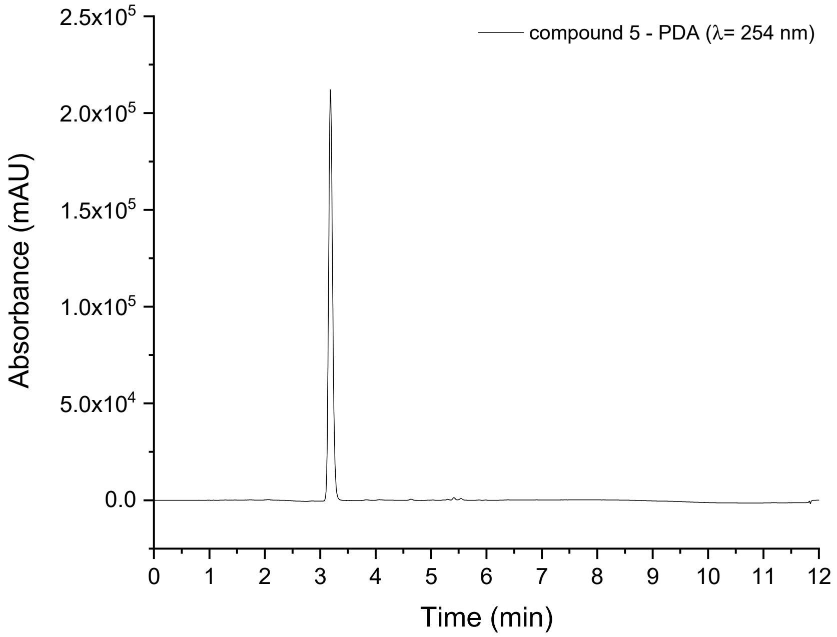

Figures S5. HPLC chromatogram for purity determination of compound **5**.

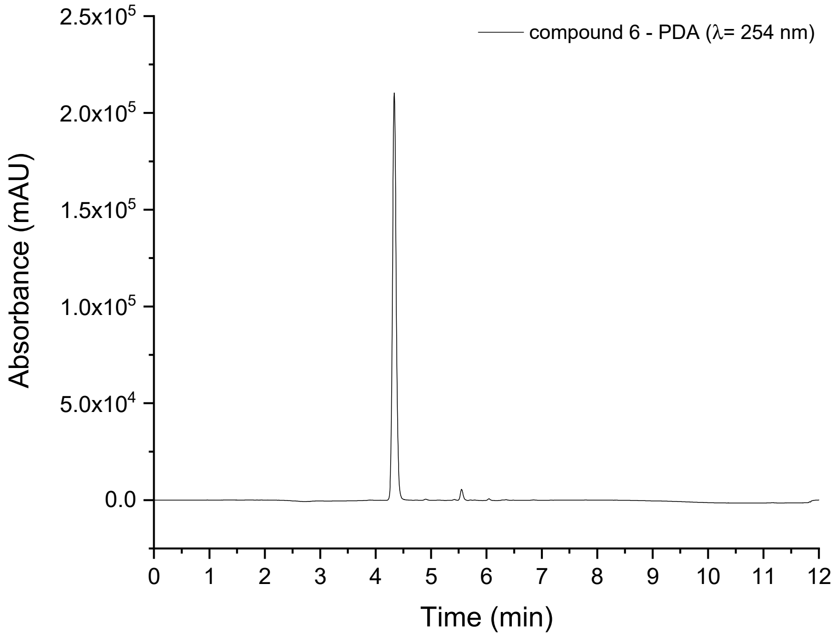

Figures S6. HPLC chromatogram for purity determination of compound **6**.

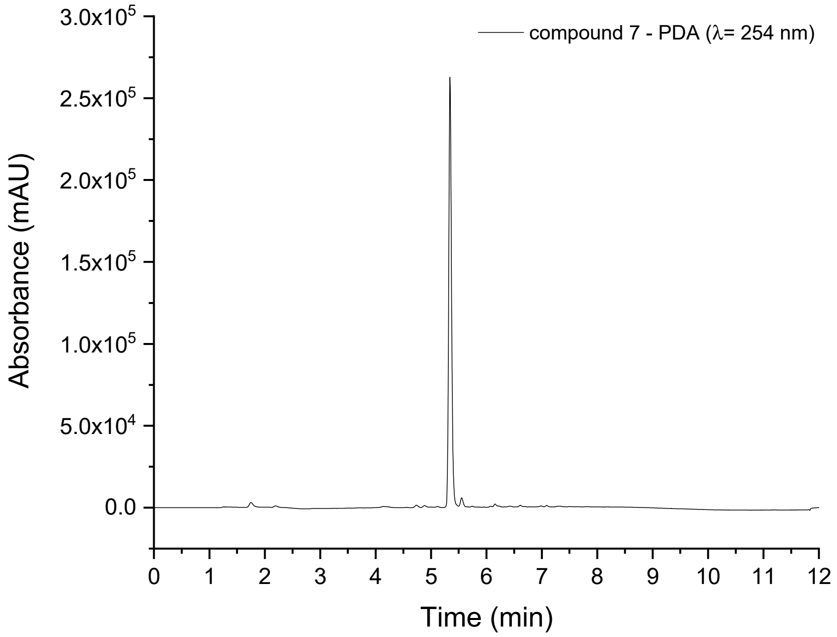

Figures S7. HPLC chromatogram for purity determination of compound **7**.

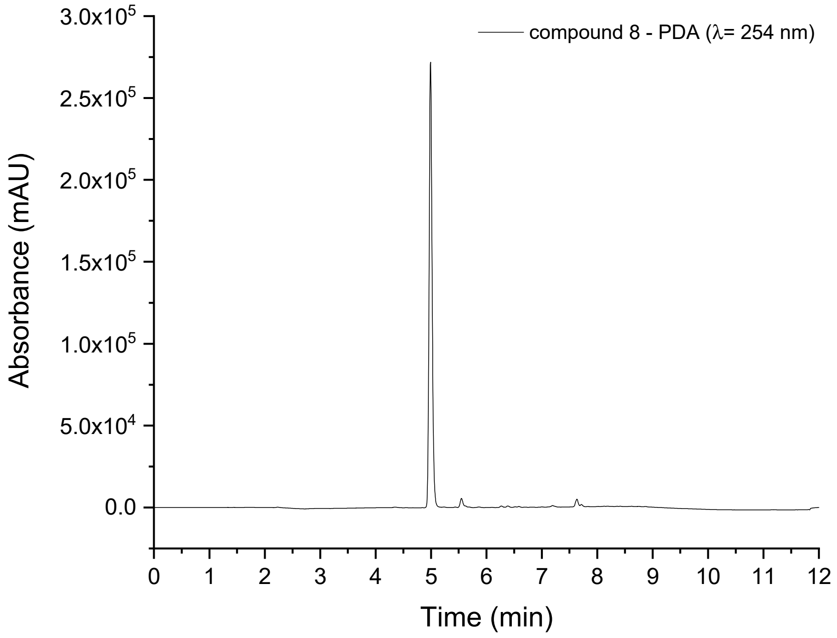

Figures S8. HPLC chromatogram for purity determination of compound **8**.

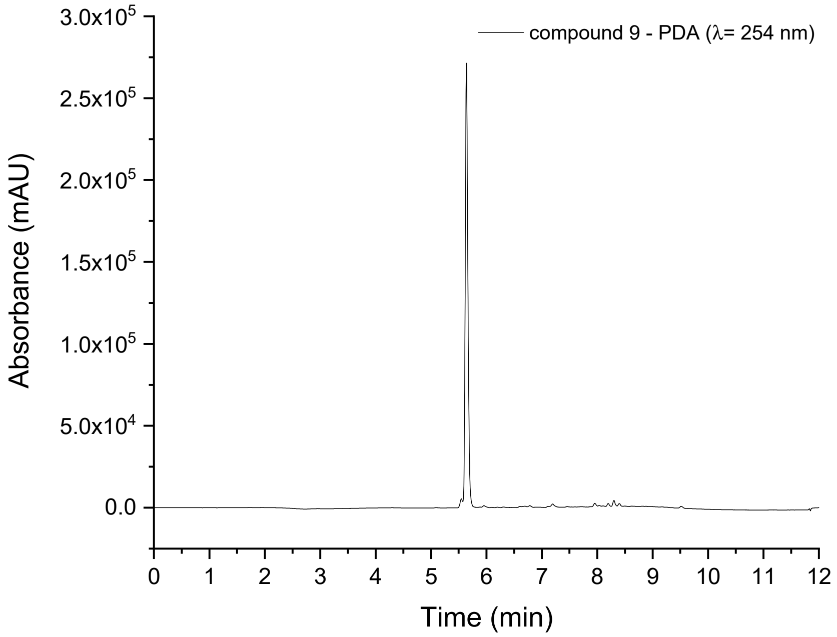

Figures S9. HPLC chromatogram for purity determination of compound **9**.

^{1}H NMR of compound 1

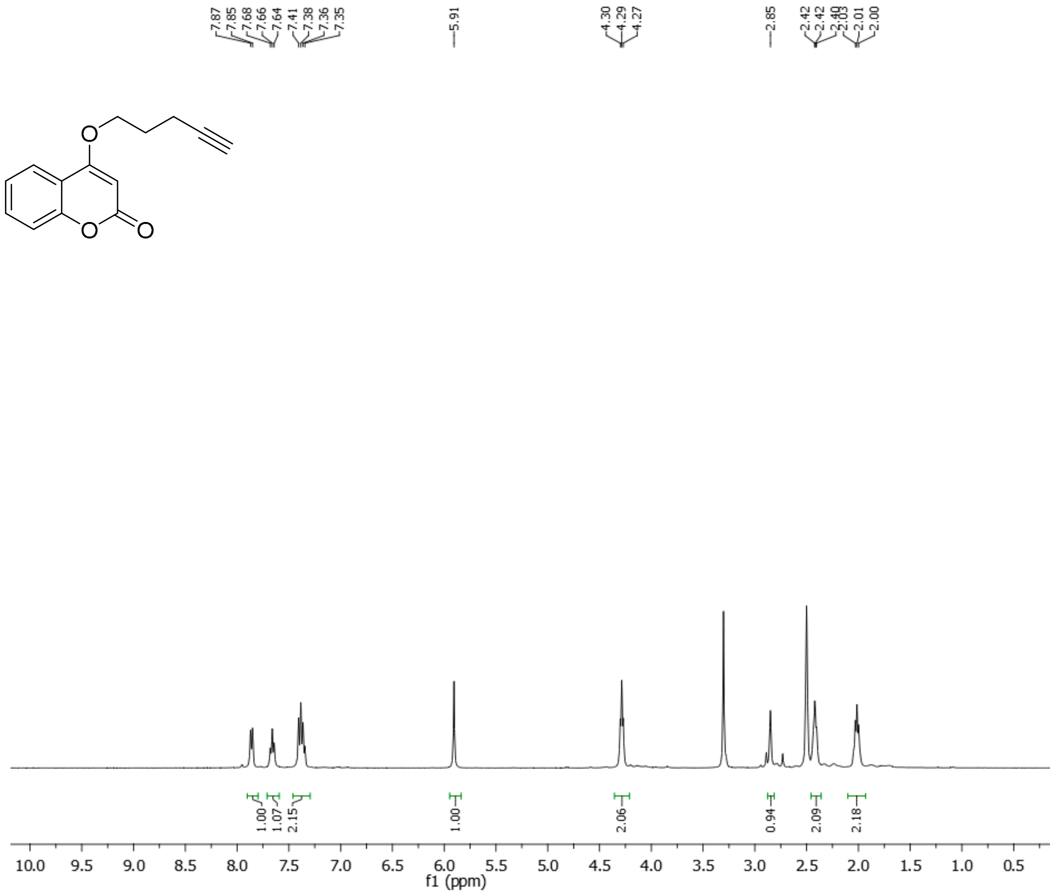

^{13}C NMR of compound 1

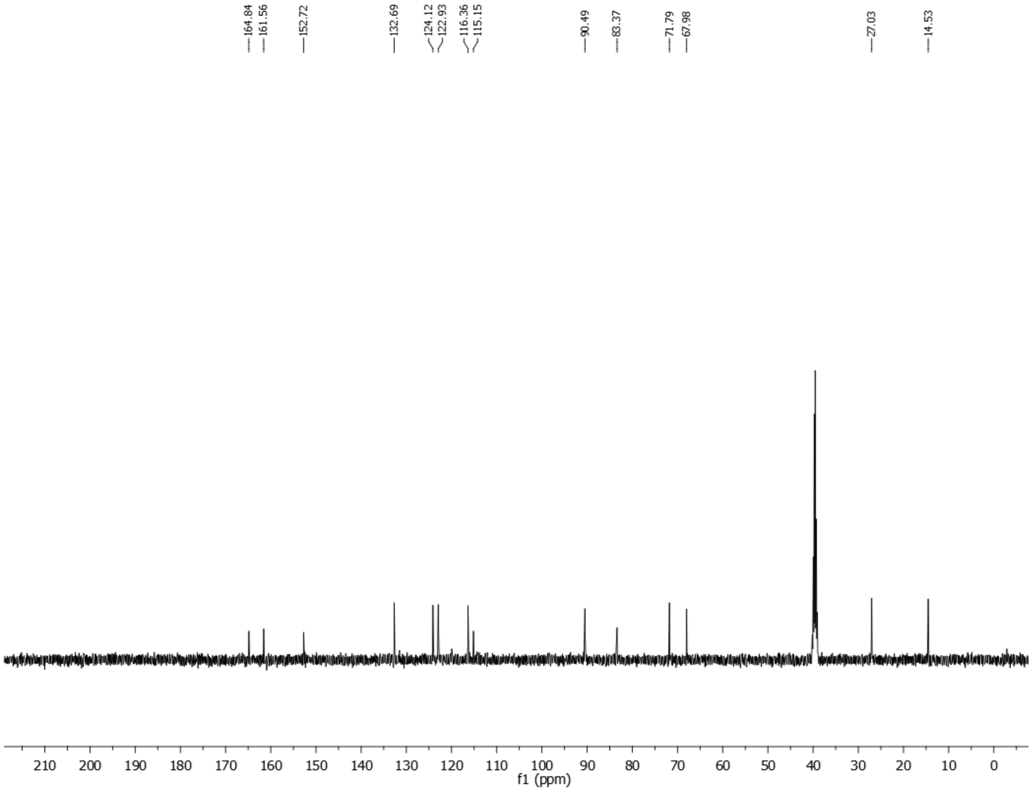

^{1}H NMR of compound 2

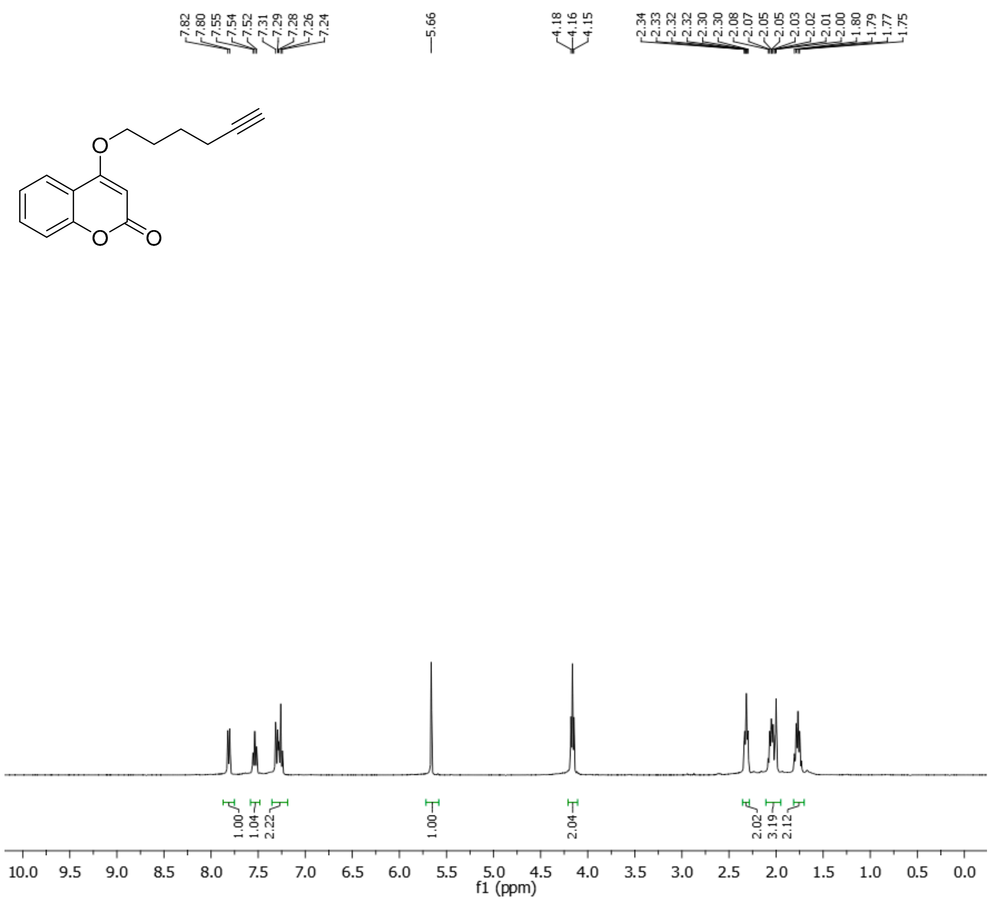

^{13}C NMR of compound 2

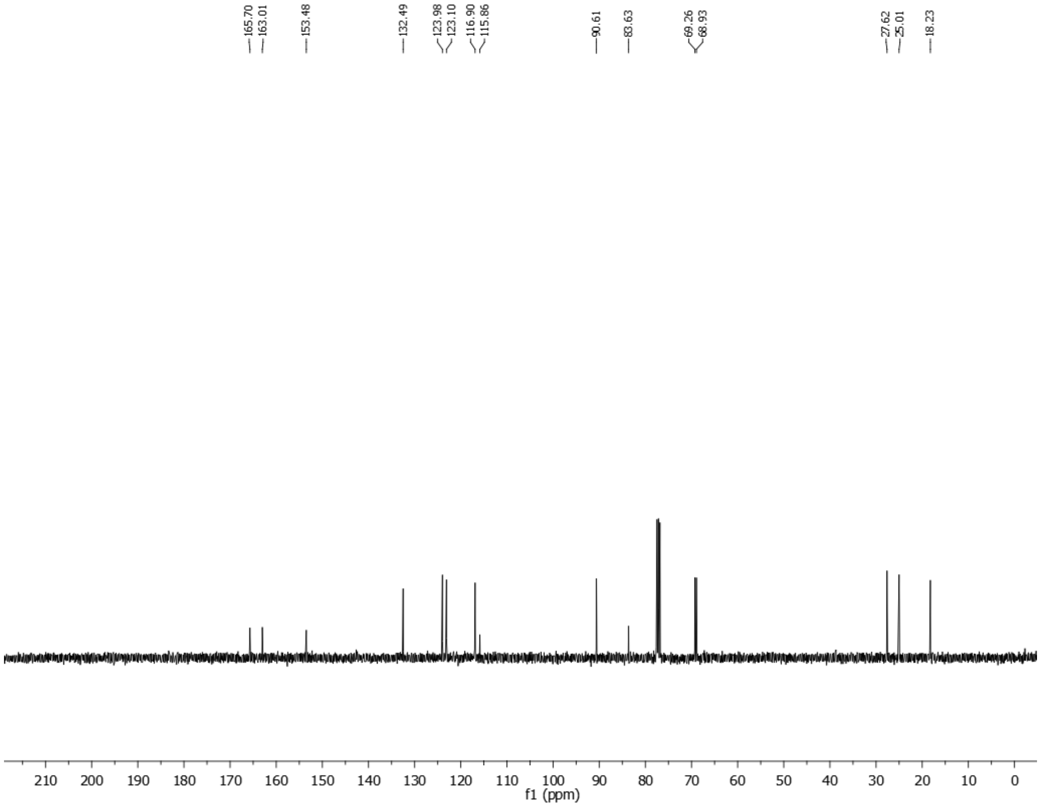

^1H NMR of compound 4

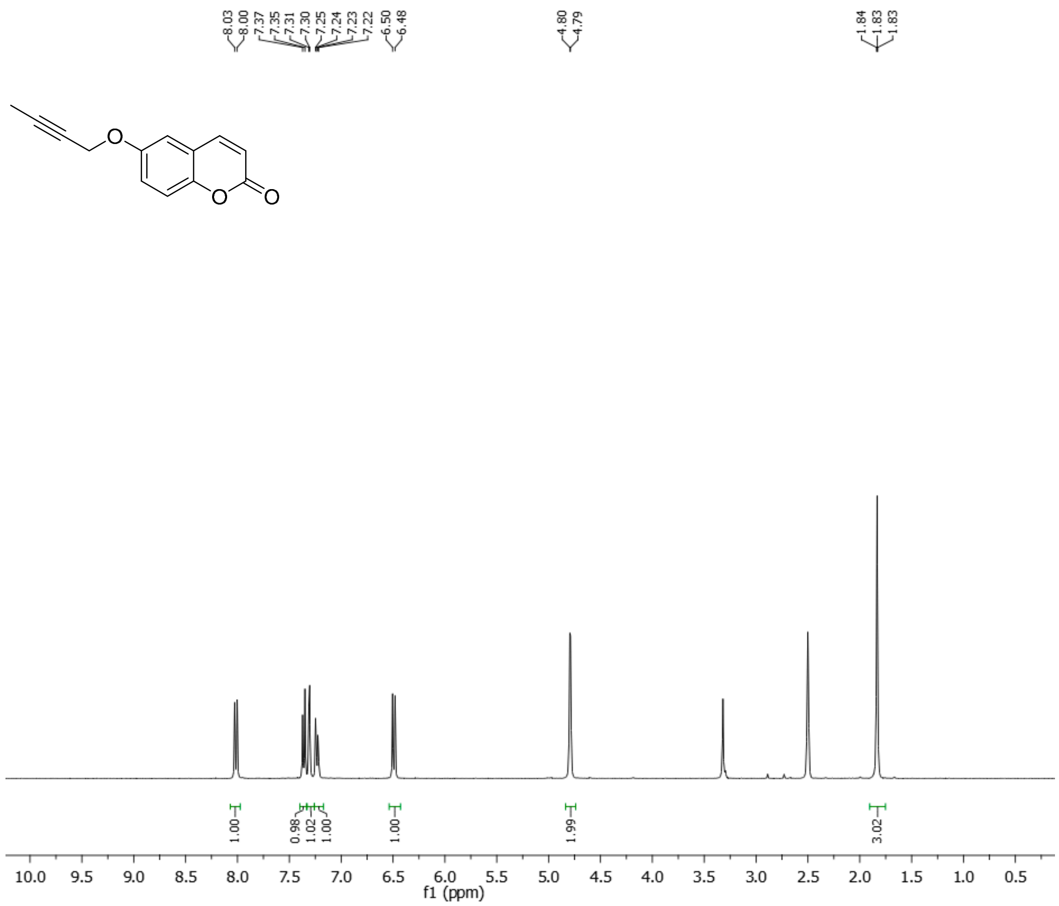

^{13}C NMR of compound 4

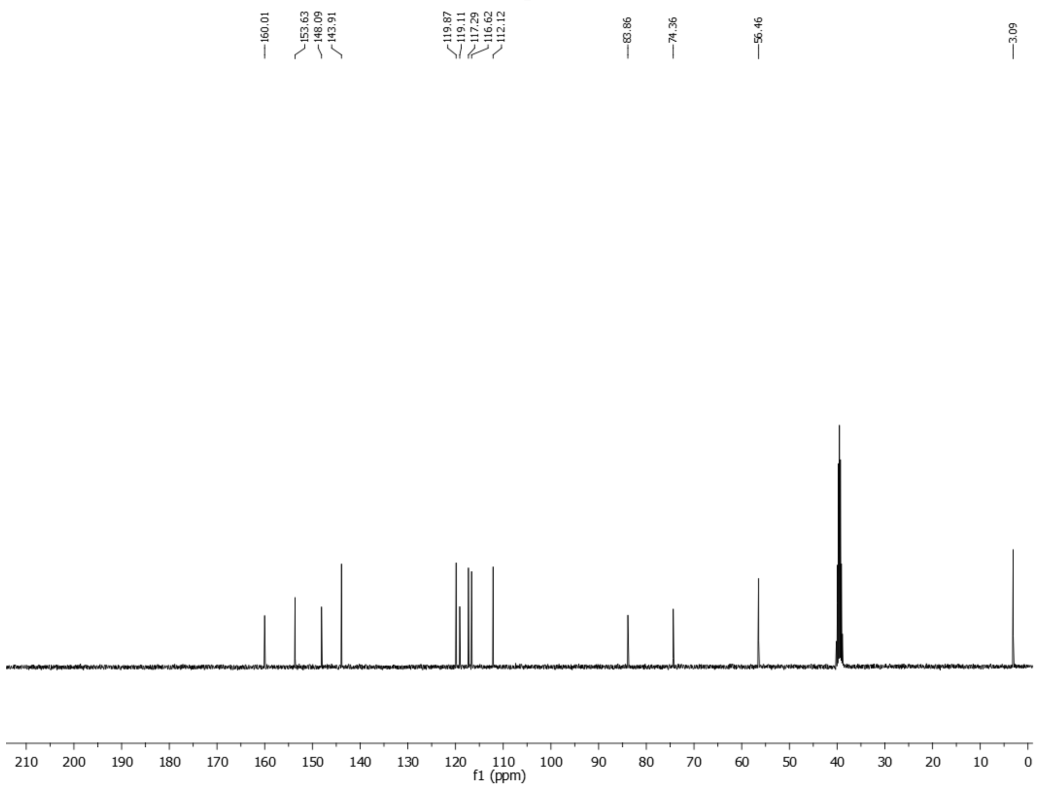

^{1}H NMR of compound 6

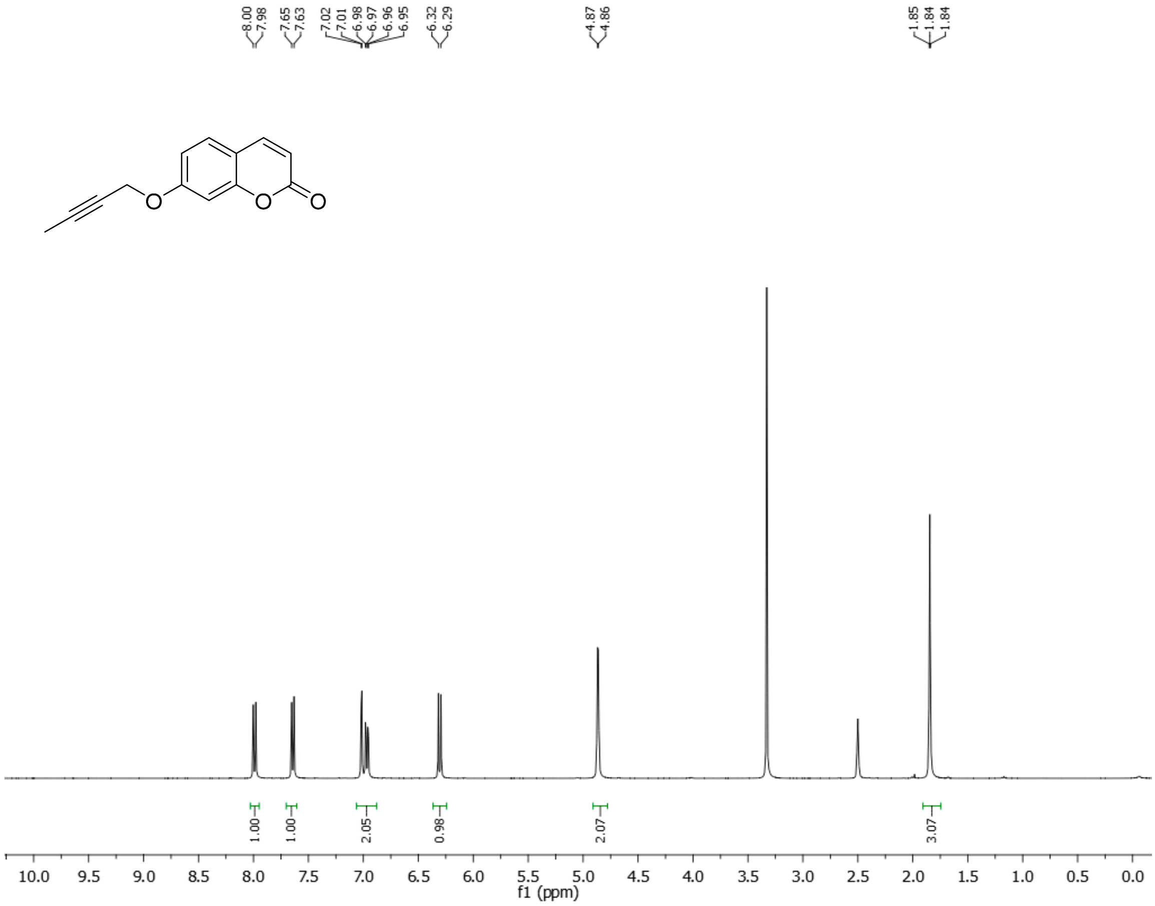

^{13}C NMR of compound 6

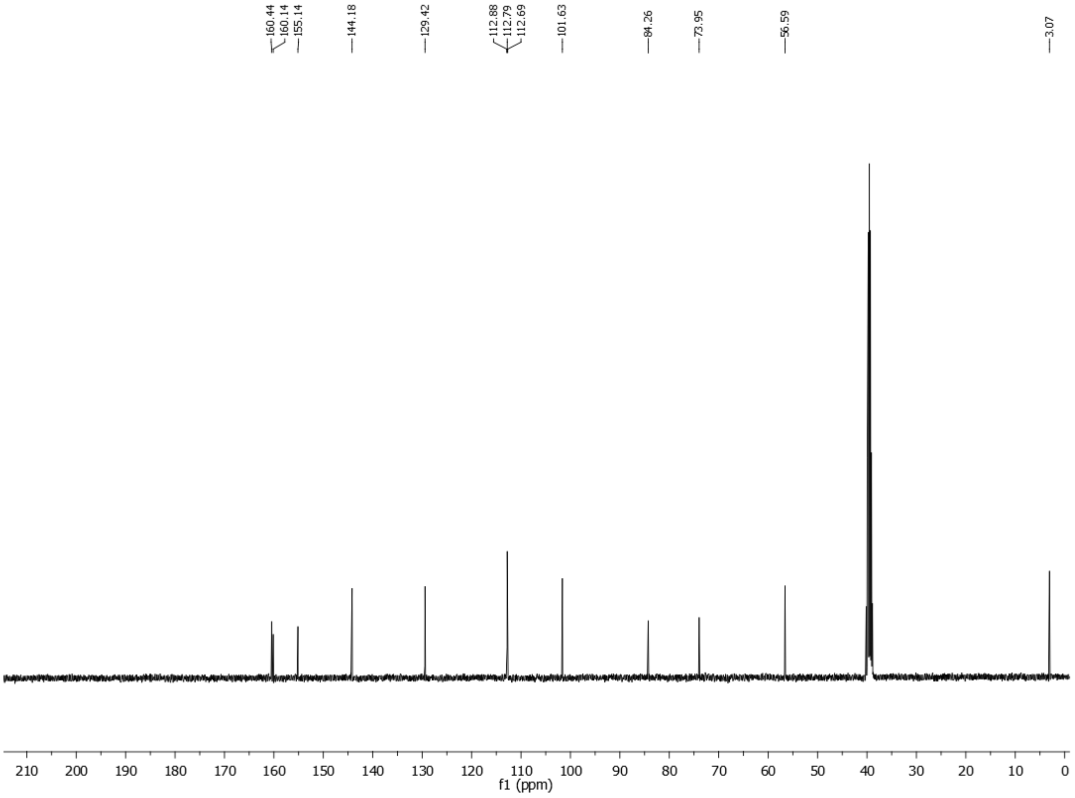

^{1}H NMR of compound 7

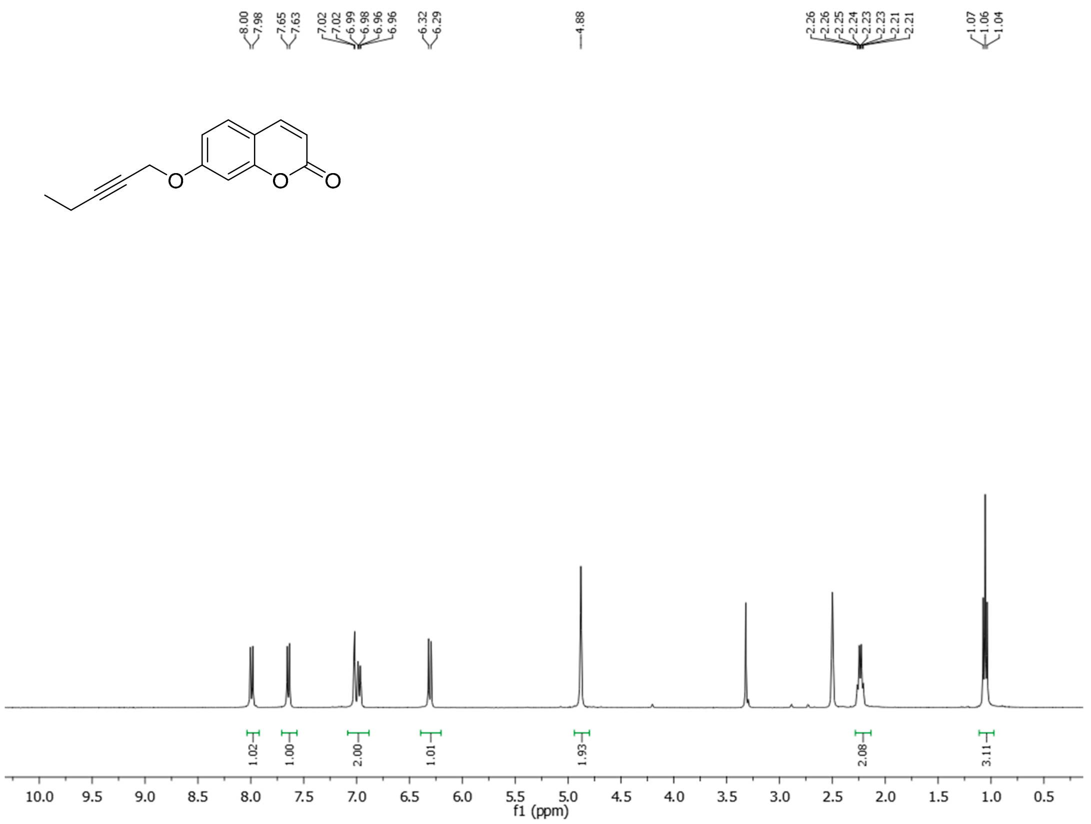

^{13}C NMR of compound 7

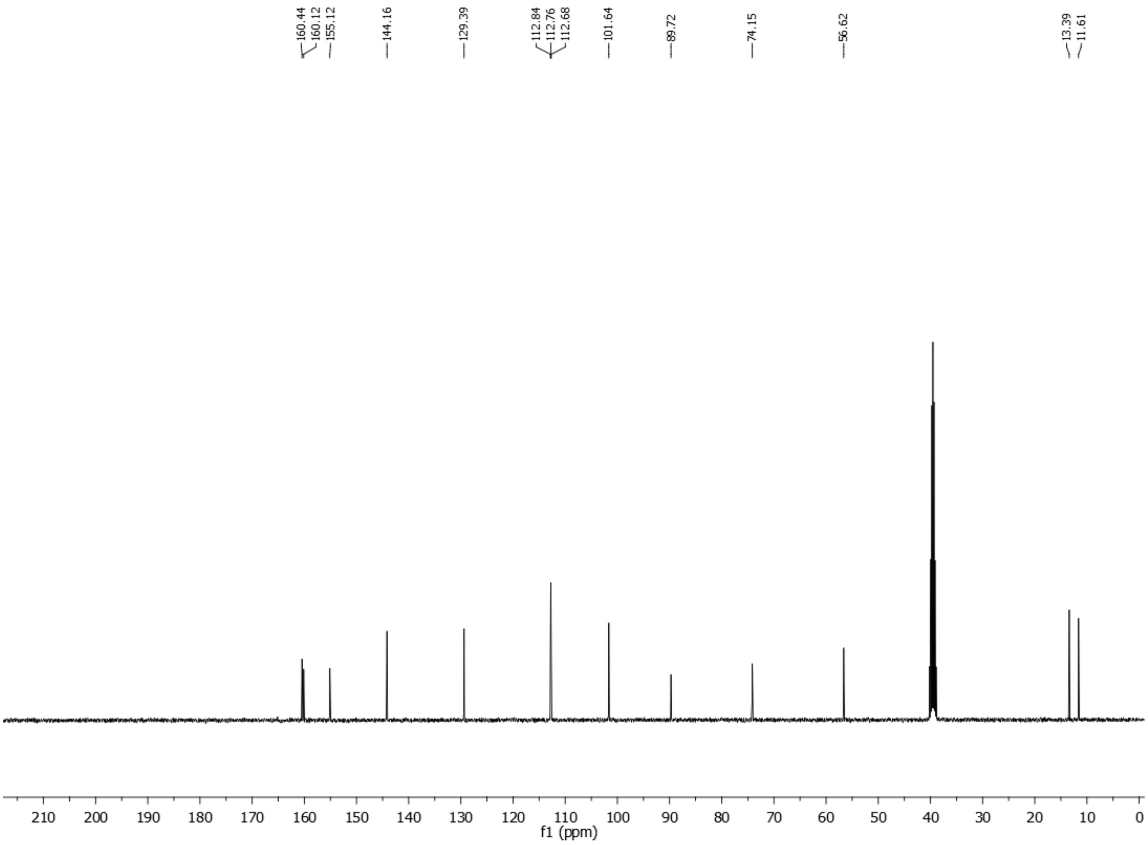

^{1}H NMR of compound 8

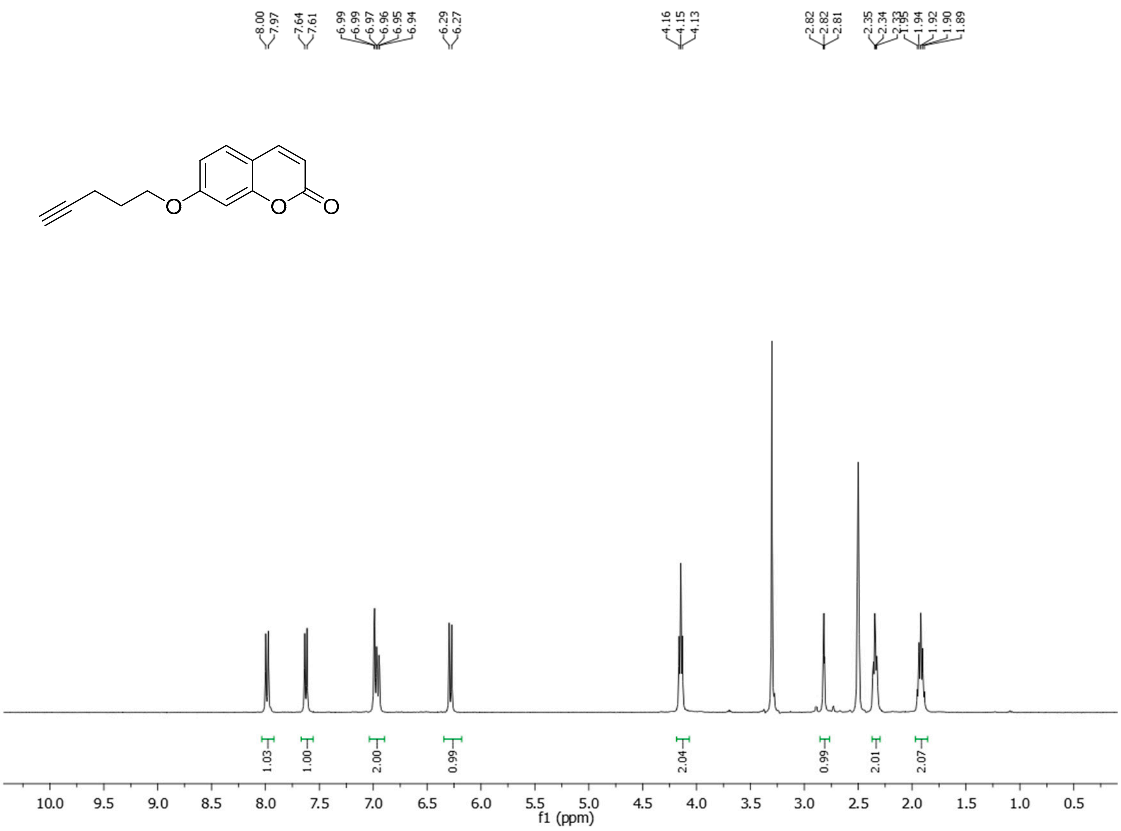

^{13}C NMR of compound 8

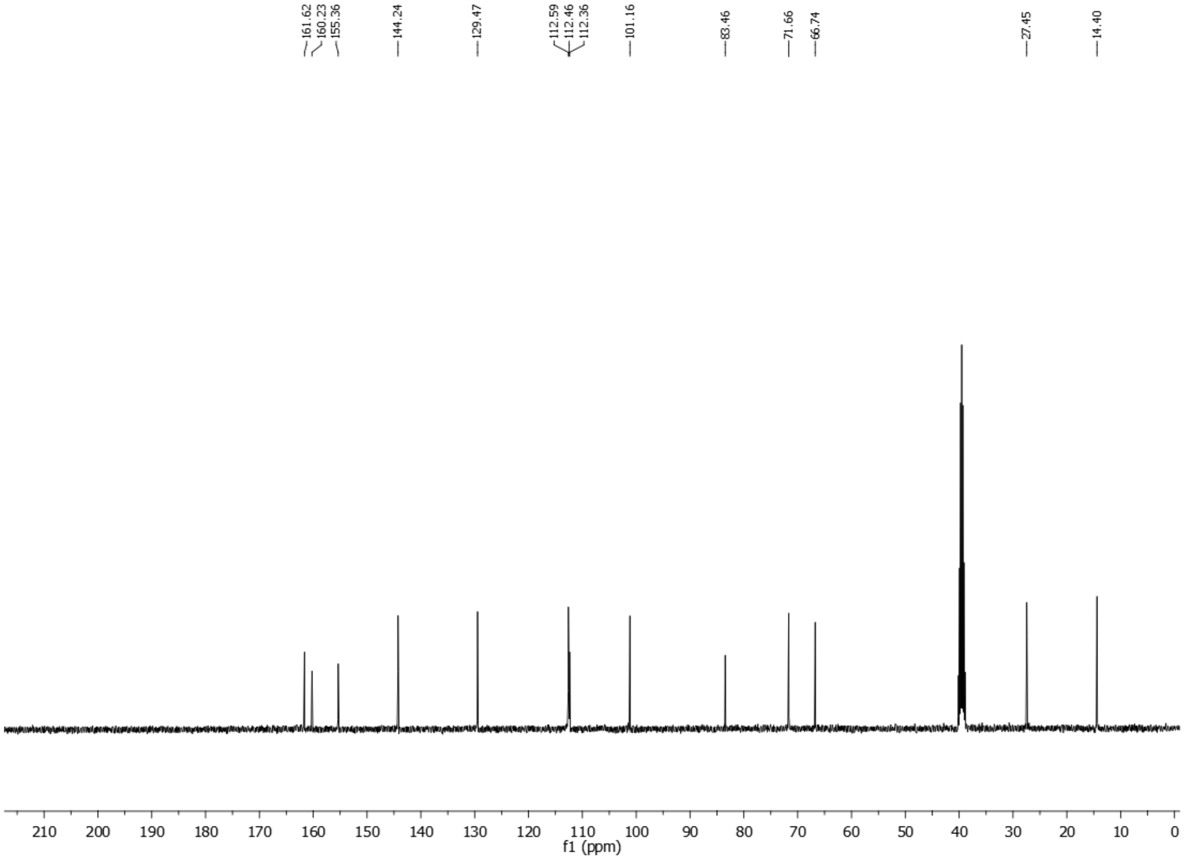

^{1}H NMR of compound 9

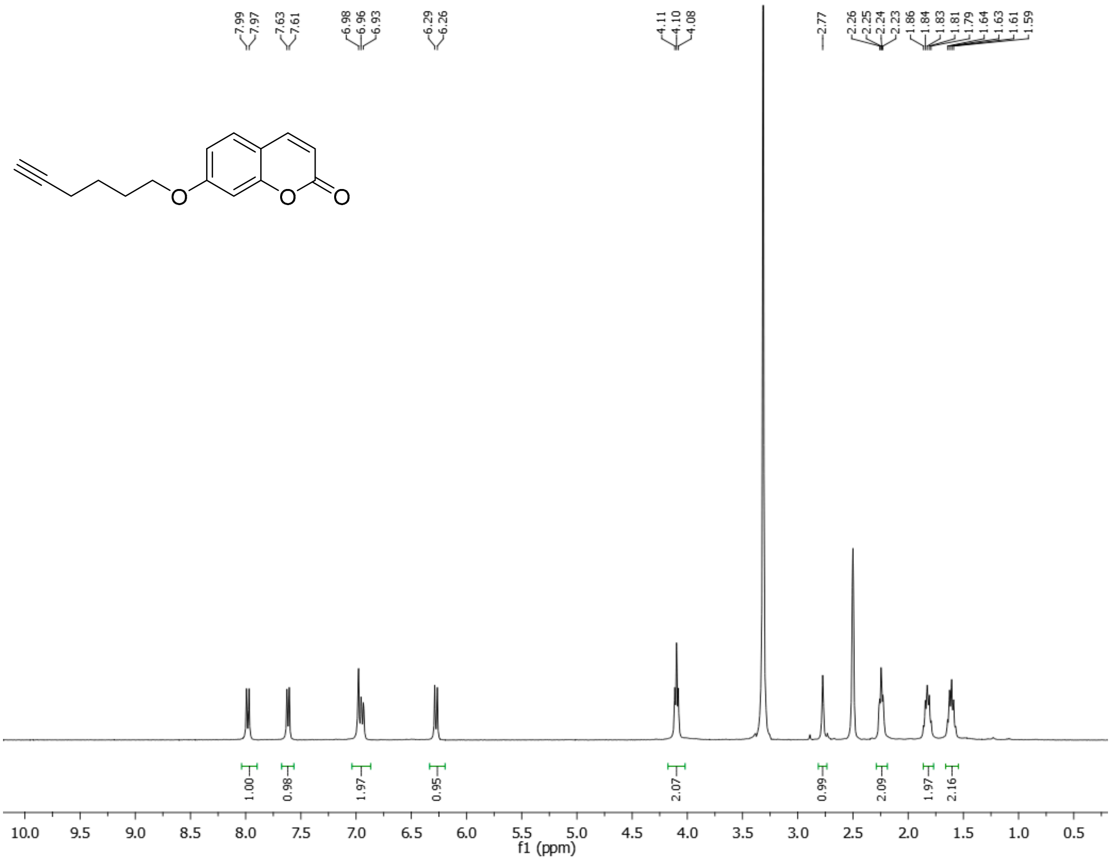

^{13}C NMR of compound 9

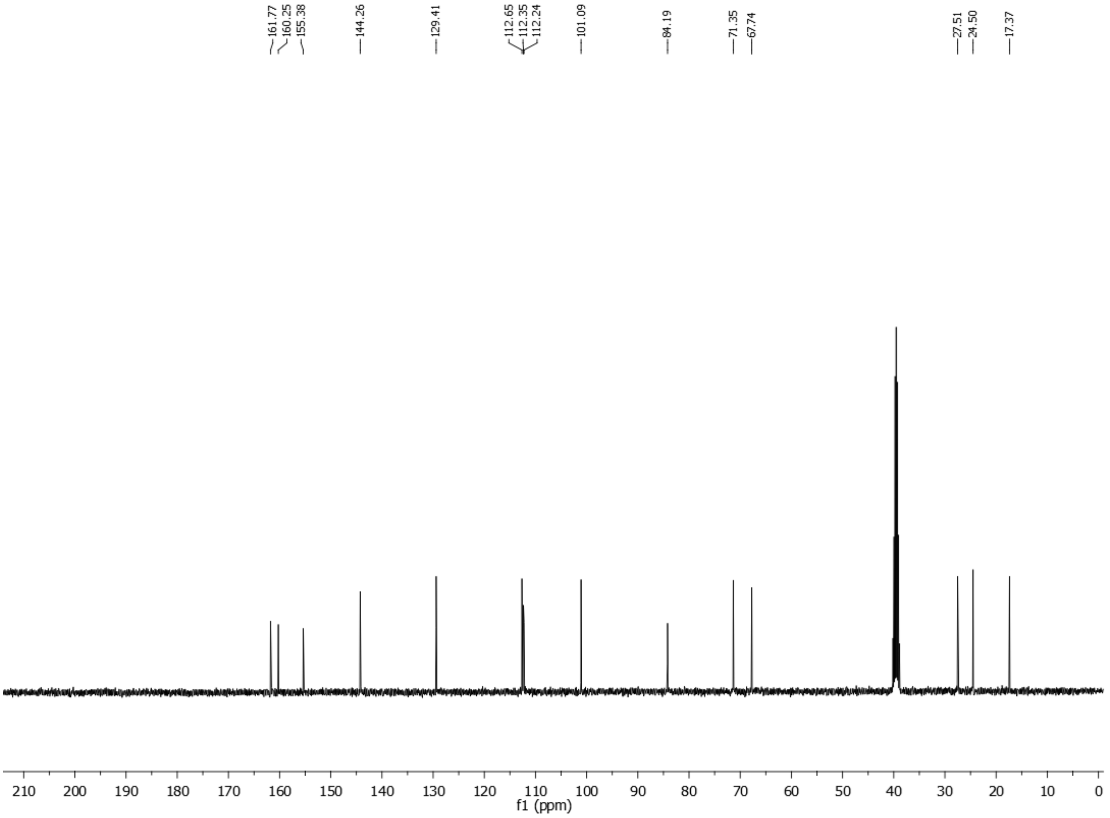

Supplement: Supplementary file 1 [file antioxidants-12-02044-s001.zip › antioxidants-2682493-SI.pdf]
